# Analytical solution of MHD bioconvection Williamson nanofluid flow over an exponentially stretching sheet with the impact of viscous dissipation and gyrotactic microorganism

**DOI:** 10.1371/journal.pone.0306358

**Published:** 2025-03-27

**Authors:** Siva Sankari, M. Eswara Rao, Awatif M.A. Elsiddieg, Waris Khan, O. D. Makinde, Taoufik Saidani, Naoufel Kraiem, Hakim AL Garalleh

**Affiliations:** 1 Department of Mathematics, Saveetha School of Engineering, SIMATS, Chennai, Tamil Nadu, India; 2 Department of Mathematics, College of Science and Humanities, Prince Sattam bin Abdulaziz University, P.O. Box 710, Al-Kharj 11942, Saudi Arabia; 3 Department of Mathematics and Statistics, Hazara University, Mansehra 21120, Khyber Pakhtunkhwa, Pakistan; 4 Faculty of Military Science, Stellenbosch University, Saldanha, South Africa; 5 Center for Scientific Research and Entrepreneurship, Northern Border University, Arar, Saudi Arabia; 6 College of Computer Science, King Khalid University, Abha, Saudi Arabia; 7 Department of Mathematical Science, College of Engineering, University of Business and Technology, Jeddah 21361, Saudi Arabia; University of Science and Technology of China, CHINA

## Abstract

Nanofluids achieve high thermal transport efficiency by uniformly dispersing small particles in base liquids, significantly enhancing the heat transfer coefficients and making them vital in various thermal engineering applications. The research examines non-uniform thermal conductivity and activation energy critical for accurately describing fluid behaviour. The study incorporates bioconvection to prevent nanoparticle settling and ensure fluid stability through motile microorganisms. The governing partial differential equations are converted into ordinary differential equations that are solved using the Homotopy Analysis Method (HAM), to provide a strong mathematical framework for the analysis. This study finds that the velocity of the fluid decreases with magnetic constraint intensification and time retardation. however, heat transfer increases at higher radiation, and heat absorption/emission parameters but decreases with a higher Prandtl number, while an increased Schmidt number leads to decreased concentration profiles. This paper investigates a nano-Williamson fluid (NWF) flow over an exponentially stretched surface in a permeable medium, considering essential variables such as mixed convection, electromagnetic forces, non-linear thermal radiation, heat production, Joule heating and ohmic dissipation that are essential for understanding its complicated behavior.

## 1.  Introduction

A non-Newtonian fluid like Williamson always stands unique in the cadre of traditional Newtonian fluids like water or air because of its varied viscosity, dependency on shear rate and applied stress. They exhibit a changing viscosity profile, unlike Newtonian fluids. They behave as solid at low shear rates with high viscous character. This transition happens at low shear rates, reducing viscosity and smooth flow. Williamson’s model shows this behavior in establishing a relationship between shear rate and shear stress. It also finds its applications where viscosity management is a key to increasing the performance of products and overall efficiency, such as in cosmetics, food processing and manufacturing. Scrutinization of bioconvection spurs in Williamson liquid by Azizs et al. [1] explains the influence of heat and motile microbes. The thermal transport features of radiative Williamson liquid are studied by Rabeeah et al. [2] on a curved exterior to analyze the effect on heat transfer dynamics because of thermal effects-liquid drift pattern bonding. The dynamics of friction effects-chemical reaction bonding on three dimensional frictional drifts in Williamson fluid is scrutinized by Moeen et al. [3]. Umair et al. [4] focussed on transporting suitable nanoparticles through blood along a curved surface. The parameters considered are Lorentz force, thermal radiation, and suction, focusing on Williamson liquid. Research provided valuable insights into this phenomenon. Khadija et al. [5] conducted a numerical analysis of non-linear radiative Casson fluids containing carbon nanotubes (CNTs) with varying lengths and radii over a permeable moving plate. Magnetohydrodynamics explores the behavior of electrically conductive fluids, such as alloys and plasmas that respond to magnetic fields. It coagulates the liquid dynamics and electromagnetism to explore the influence of liquid behavior on the magnetic field and vice versa. Its applications are varied, ranging from celestial astrophysics to spacecraft propulsion systems. It also contributes to geophysics, atmosphere and oceans, focusing on interactions with the earth’s magnetic field. Some appreciable features involve energy conversion and fluid control through pumps and generators, offering the most efficient and reliable solutions with minimal error and moving parts. Avinash et al. [6] discovered the inspiration of magnetic fields on bioconvection drift, in which the connection between magnetic fields and microorganisms in bioconvection is explored in detail. Makinde et al. [7] explored the phenomenon of bioconvection in nanofluid in the horizontal phase of paraboloid, summarising the impression of thermophoresis and Brownian motion accompanied by non-linear thermal emission and quartic chemical process. The thermal complexities of MHD Williamson liquid drift within a microchannel are studied by Shashikumar et al. [8], focused on heat transference dynamics and the magnetic field impressions over liquid drift patterns. Heat and mass transference in MHD Williamson nano liquid drift over a stretched exterior is explored by Kashif et al. [9], focussing on the inspiration of magnetic field on thermal energy and particle diffusion to understand the thermal effects and mass transfer in nano liquid drift. Musharafa et al. [10] analyze the influence of radiation on the behavior of magneto cross-nano Williamson liquid on exponentially extending sheets. This non-linear radiation changed the conventional heat transference, while Stefan blowing induces blowing or suction effects that impact drift dynamics. Aisha et al [11] conducted a detailed study of magnetohydrodynamic (MHD) rotating flow over a stretching surface, concentrating on how viscosity and the clumping of nanoparticles affect the flow. Zafar et al. [12] explored the significance of the shape factor on magnetohydrodynamic buoyancy-driven thin film flow of nanofluid over an inclined sheet with slip conditions, along with an analysis of irreversibility. The impact of non-linear radiation and Stefan blowing, a novel and intriguing area of research, is deliberated by Siva Sankari et al. [13], concentrating on the behavior of magneto-cross Williamson liquid through an exponentially extending sheet. Radiation alters heat transference, whereas Stefan blowing impacts the liquid boundary by blowing or suction. Studies on intricate dynamics of liquid drift of rotating MHD Williamson nanofluids in three-dimensional space are studied comprehensively by Muhammad et al. [14]. Numerical inquiries on the double diffusion heat model cascading over an exponentially stretched surface are explored by Amjad et al. [15], involving scrutinizing the conduct of nanofluids, emphasizing the interplay between surface and thermal conductivity. Zafar et al. [16] studied heat transfer in radiative hybrid nanofluids over a moving sheet with porous media and slip conditions, focusing on the numerical analysis of variable viscosity and thermal conductivity. Zafar et al[17] analyzed hybrid nanofluid’s mixed convective stagnation point flow over a sheet with variable thermal conductivity and slip conditions through a model-based study. The concept of viscous dissipation, which involves mechanical to heat energy conversion within a liquid surface, is more than just a theoretical construct. It plays a crucial role in heat transport and directly impacts the efficiency of pumps, heat exchangers, and other engineering systems. The management of dissipation is a vital step for high efficiency and precise predictions in these systems. Our research underscores the practical implications of understanding and managing dissipation, highlighting its potential to improve real-world application performance significantly. Salahuddin et al. [18] made a comprehensive numerical analysis of altering nanofluids, prioritizing the thermal and solutal drift dynamics involving viscous dissipation. Moeen et al. [19] investigated 3-dimensional drift qualities such as viscous dissipation, overlooking the influences of activation energy and radiation on liquid drift. Viscous dissipation in Williamson liquid over a horizontally saturated porous plate with fixed wall temperature was studied by Hussein et al. [20]. This study also covers the impact of liquid viscosity on dissipation and interaction with porous medium and thermal gradient. An in-depth examination of the dictating drift dynamics of MHD heat and mass transference is initiated by Sina et al. [21] on a porous cylinder. With its comprehensive approach, this research sheds light on the interplay between magnetic fields and chemical reactions within the liquid surface. Khadija et al. [22] discussed the entropy analysis of the Hall effect on rotating hybrid nanofluid flow over a nonlinear radiative surface, considering variable viscosity and slip conditions. Umar et al. [23] studied the significance of the unsteady rotating flow of nanofluid, focusing on nanoparticle aggregation, slip conditions, and the effects of variable viscosity. Joule’s law, which involves converting electrical to heat energy, is supported by the interaction between atoms and electrons. It finds its applications in widespread devices such as toasters and industrial appliances. Incompetent output in electrical systems and energy loss are plausible if the energy management is not up to the standards. The outcome of radiation & Joule heating on MHD Williamson nano liquid is deliberated by Kumar et al. [24], including the factor of entropy generation. The study yielded valuable insights for understanding the nanofluid systems in heat transference and its consequential impact on various fields, such as thermal engineering. Ijaz Khan et al. [25] explored entropy optimization in Williamson nanofluid drift by theoretical modeling, computational analysis and experimental analysis to better understand the system dynamics. Numerical simulations are carried out in a porous medium by Amir Abbas et al. [26] to investigate the Magnetohydrodynamics of a dissipating Williamson-Sakiadis drift, focusing on the drift behavior kindled by solar radiation and Joule heating. Khadija et al. [27]conducted a numerical investigation into the entropy generation due to Joule heating in the non-axisymmetric flow of hybrid nanofluid over a stretching surface.The combined effect of mixed convection of Williamson liquid with suction/blowing is investigated by Majid et al. [28] over a permeable wedge, considering viscous dissipation and heat as parameters. Williamson liquid exhibits viscoelastic characteristics, whereas the permeability of the wedge plays a vital role in the drift dynamics due to buoyance and various factors that affect the shape drift. Pankaj et al. [29] worked on the drift of MHD Williamson micropolar liquid through an extending pane inspired by liquid drift and heat transfer. This study adds value to understanding complex liquid systems and their applications. Modeling and examination of nanofluid over an extended sheet are scrutinized by Jifeng et al. [30], focussing on liquid behavior and heat transfer dynamics. Understanding thermal radiation properties is a mandatory path to explore various avenues in engineering, material science, astronomical science, etc. Bioconvection within MHD nano liquid drift through a paraboloid revolution was studied by Makinde et al. [31], drawing inspiration from thermal radiation and quartic autocatalysis chemical reactions and their influence on liquid dynamics and magnetic fields. Anagandula et al. [32] worked on the inspiration of radiation and inclined magnetic field on Williamson liquid drift, considering velocity and thermal slips. This research plays a vital role in underscoring the complexity due to alterations in thermal distribution in magnetic and radiation fields. Fuzhang et al. [33] deliberated the influence of radiation and magnetic fields in Williamson liquid drift over an extended sheet. Bioconvection is an exciting area that correlates biology, liquid dynamics and environmental science. The grasp of biological systems and innovations is primarily due to delving into the mechanism. Puneeth et al. [34] worked on unveiling the intricate dynamics of bioconvection observed in hybrid nanofluid, dwelling on various interplay factors such as nanoparticle behavior and chemical reactions. Muhammad et al. [35] explored the characteristics of Williamson ferro-nanofluids exposed to bioconvection, considering the parameters of energy variations and magnetic dipole effects. Zubair et al. [36] Research on chemically reactive bioconvection in three-dimensional drift dynamics showed the oxytactic character of nanofluid-containing microorganisms. Aqsa et al. [37] explored bioconvection within an MHD Prandtl nanofluid in a gyrotactic microbial environment paved the way for potential biofuel usage from microbes. Activation energy is the lowest energy essential to kick-start a chemical reaction. It is modeled as a potential constraint that the molecules should overcome to enter the phase of product creation. Eswara Rao et al. [38] performed a detailed analysis of the performance of activation energy in MHD stretched drift of Williamson and Casson nano liquid through a permeable medium. This study focussed on the transport phenomena of nanofluids, combining the effects of both activation energy and bioconvection. Research on activation energy focussing on chemical kinetics-magnetic field interactions done by Asjad et al. [39] involves monitoring the variation of characteristics of nanofluid by varying activation energies. Shoaib et al. [40] deliberated the impact of activation energy of chemical reactant drift induced by entropy on a Williamson nanofluid. Xianqin et al. [41] conducted numerical simulations on vertical elongating pipes to explore the radiative drift of Williamson nanofluid, incorporating variations of activation energy to the system in the presence of swimming microbes. Motile microbes move without external influence, using pseudopodia and flagella as extensions. These organisms find their homes in terrestrial soil, aquatic zones and the human body. Khan et al. [42] worked on MHD nano liquid drift through a heat-elongating pane in the presence of gyrotactic microbes in the magnetic field to better understand microfluidic systems. Gyrotactic microorganisms in radiative Williamson nanofluid drift in the existence of ferromagnetic particles are explored by Mostafa et al. [43] to explore their influence on the drift of nanofluids. Bhatti et al. [44] comprehensively analyzed the characteristics of gyrotactic microorganisms swimming inside the MHD nanofluid drift flows among revolving spherical plates in a permeable medium. The dynamic behavior of microorganisms in the human bloodstream is explored by Rana et al. [45], focussing on movement in a nano bioconvective Williamson fluid to enhance bio-convection.

Analytical solution of MHD bioconvection Williamson nanofluid flow over an exponentially stretching sheet with viscous dissipation and gyrotactic microorganisms presents several novel contributions: Bioconvection is combined with magnetohydrodynamics (MHD) in Williamson nanofluids, providing a new look into fluid dynamics under magnetic fields, which had not been studied widely before. It studies fluid flow over an exponentially stretching sheet used in materials processing, addressing complex behaviors usually ignored in simpler models. Viscous dissipation is discussed to illustrate how kinetic energy changes to thermal energy, which is vital for understanding heat transfer in nanofluids. The impact of gyrotactic microorganisms on fluid dynamics is considered to reflect biological and environmental situations. The use of HAM for solving complex non-linear equations provides analytical insights that go beyond numerical methods. The paper offers an elaborate study on heat and mass transfer incorporating factors such as Brownian motion and thermophoresis, which are important in optimizing the engineering applications of nanofluids. Current findings have various applications in many fields, including controlled drug delivery systems, chemical reactors, heat exchangers, and steam generators.

### Research objective

To make the introduction of the paper more appealing, the following research objective questions can be added. These questions are meant to be brief, discerning and directly tied to the analysis and discussion of findings provided in this study.

1. What is the effect of gyrotactic microorganisms on Williamson nanofluid’s thermal and mass transport properties?
2. What is the effect of varying magnetic field strengths on velocity and temperature profiles for nanofluid flow across an exponentially stretching sheet?
3. How does overall heat transfer efficiency in the system change due to activation energy and viscous dissipation, respectively?
4. To nanofluids, explain how Brownian motion and thermophoresis interact, affecting their stability and performance.

### Practical applications

There are many practical applications in different fields for the study of MHD bioconvection in Williamson nanofluid flow over exponentially stretching sheet. The study further indicates that heat exchangers and cooling systems can be improved by having nanofluids with enhanced thermal conductivity particularly when magnetic fields are used to optimize heat transfer. In biotechnology, gyrotactic microorganisms are incorporated into bioreactor design to enhance microbial drift and concentration. It also deals with viscous dissipation as well as joule heating in energy conversion systems thus increasing efficiencies of such devices like pumps and generators. Environmental applications benefit from understanding bioconvection, leading to more efficient wastewater treatment methods. Plasma behavior in space is aided by exploring magnetohydrodynamics through this research which assists in spacecraft propulsion and space technology development. Finally, interaction between nanoparticles and base fluids provides a way to tailor thermal properties making them an essential ingredient for electronics and aerospace industries among others.

### 2.  Mathematical formulation

**Fig 1:**
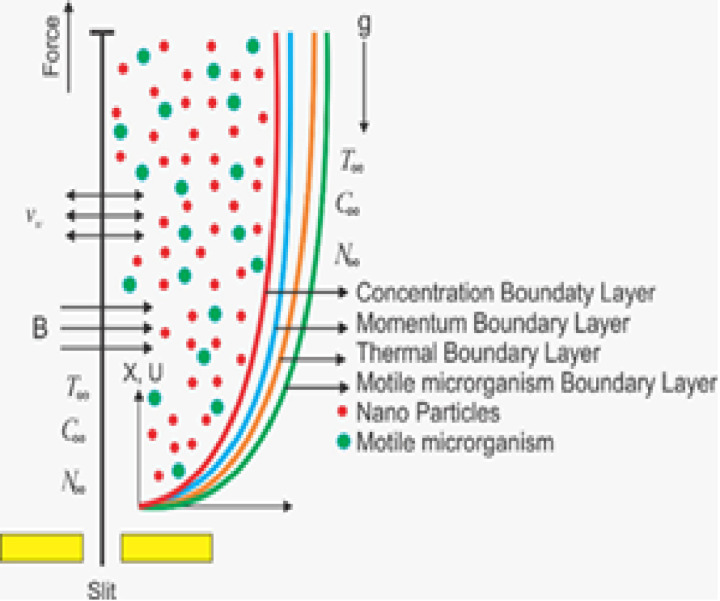
Diagram of the flow model.


The study investigates a steady drift of two-dimensional Williamson liquid initiated by a sheet extending exponentially and embedded within a porous medium. Terms related to heat absorption/emission and thermal radiation are included in the energy equation. It also considers phenomena like Brownian motion and thermophoresis. Additionally, activation energy is considered to influence mass transference. The composition of nanofluid remains unaffected by gyrotactic mobile microorganisms. The equation is adjusted to integrate the influences of joule heating and ohmic dissipation mechanisms while accounting for activation energy and bioconvection inspirations. The function signifies the velocity of the extending sheet $U_{w}=a_{0}e^{\frac{x}{l}}$, where$a_{0}$ and *l* are constants determining the sheet’s elongating rate and length scale, respectively. A schematic view of the current drift is delivered in Fig 1, illustrating the spatial distribution and directionality. The velocity of the liquid for two-dimensional drift is given by u and v. These equations describe how the model works [10,39]:

$$u_{x}+v_{y}=0$$ (1)

$$\left.\begin{array}{l} uu_{x}+vu_{y}=vu_{yy}+\sqrt{2}u_{y}u_{yy}-\frac{\sigma }{\rho }\left(B_{0}^{2}u\right)-\frac{\upsilon }{k_{1}}u\\ +\frac{1}{\rho }\left[g\beta_{\rho }(1-C_{\infty })(T-T_{\infty })-g(\rho_{p}-\rho_{f})(C-C_{\infty })-g\gamma (\rho_{m}-\rho_{f)})(N-N_{\infty })\right] \end{array}\right\}$$ (2)

$$\left.\begin{array}{l} uT_{x}+vT_{y}=\alpha T_{yy}-\frac{1}{\rho C_{p}}\frac{\partial q_{r}}{\partial y}+\tau \left(D_{B}T_{y}C_{y}+\frac{D_{T}}{T_{\infty }}{\left(T_{y}\right)}^{2}\right)\\ +\frac{\gamma }{c\rho }\left(1+\frac{\Gamma }{\sqrt{2}}{\left(\frac{\partial u}{\partial y}\right)}^{2}\right)1+\frac{\Gamma }{\sqrt{2}}{\left(\frac{\partial u}{\partial y}\right)}^{2}+\frac{Q_{0}}{\rho C_{\rho }}(T-T_{\infty })+\frac{\upsilon }{k_{1}C_{p}}u^{2}+\frac{\sigma B_{0}^{2}u^{2}}{\rho C_{p}} \end{array}\right\}$$ (3)

$$uC_{x}+vC_{y}=DC_{yy}-K_{r}^{2}(C-C_{\infty }){\left(\frac{T}{T_{\infty }}\right)}^{n}\exp\left(\frac{-Ea}{kT}\right)+\frac{D_{T}}{T_{\infty }}T_{yy}$$ (4)

$$uN_{x}+vN_{y}+dW_{c}\frac{\partial }{\partial y}\left(\frac{N}{\Delta C}C_{y}\right)=N_{yy}D_{n}$$ (5)

Adhering to the Roseland approximation [7,31] for the radiative heat flux, where k * represents the average absorption coefficient and σ* denotes the Stefan-Boltzmann constant.


$$\left.\begin{array}{l} uT_{x}+vT_{y}=T_{yy}\left(\alpha +\frac{16\sigma^{*}T_{\infty }^{3}}{\rho C_{p}3k_{1}}\right)+\tau \left(D_{B}T_{y}C_{y}+\frac{D_{T}}{T_{\infty }}{\left(T_{y}\right)}^{2}\right)\\ +\frac{\gamma }{c\rho }\left(1+\frac{\Gamma }{\sqrt{2}}{\left(\frac{\partial u}{\partial y}\right)}^{2}\right)1+\frac{\Gamma }{\sqrt{2}}{\left(\frac{\partial u}{\partial y}\right)}^{2}+\frac{Q_{0}}{\rho C_{\rho }}(T-T_{\infty })+\frac{\upsilon }{k_{1}C_{p}}u^{2}+\frac{\sigma B_{0}^{2}u^{2}}{\rho C_{p}} \end{array}\right\}$$ (6)

The corresponding boundary conditions are outlined below:


$$\begin{array}{l} u=U_{w},v=-\gamma (x),T=T_{w},C=C_{w,}N=N_{w}aty\to 0\\ u\to 0,T\to T_{\infty },C\to C_{\infty },N\to N_{\infty }asy\to \infty \end{array}$$ (7)

The subsequent similarity transformation group transforms governing equations[39].


$$U_{w}=a_{o}e^{x/l},\gamma (x)=-V_{0}e^{x/2l},T_{w}=T_{\infty }+T_{0}e^{x/2l},C_{w}=C_{\infty }+C_{0}e^{x/2l},N_{w}=N_{\infty }+N_{0}e^{x/2l}$$ (8)

$$\left.\begin{array}{l} \eta =y\sqrt{\frac{a_{0}}{2\upsilon l}e^{x/2l}},u=a_{0}e^{x/2l}f'(\eta ),v=\sqrt{\frac{a_{0}}{2l}}e^{x/2l}[f(\eta )+\eta f'(\eta )],\psi =\sqrt{2la_{0}}f(\eta )e^{x/2l},\\ T=T_{\infty }+T_{0}e^{x/2l}\theta (\eta ),C=C_{\infty }+C_{0}e^{x/2l}\phi (\eta ),N=N_{\infty }+N_{0}e^{x/2l}\chi (\eta ) \end{array}\right\}$$ (9)

Given the above suitable relations, equations (1) are satisfied identically and equations (2)-(5) consistently develop


$$f'''-(M+K_{p})f'-2f{'}^{2}+ff''+Wf''f'''+\lambda (\theta -Nr\phi -Rb\chi )=0$$ (10)

$$\left.\begin{array}{l} \theta ''\left(1+\frac{4}{3}K\right)+\mathrm{Pr}f\theta '-\mathrm{Pr}\theta f'+\mathrm{Pr}\theta '\left(Nb\phi '+Nr\theta '\right)+\mathrm{Pr}Ec{\left(f''\right)}^{2}+\\ \mathrm{Pr}\frac{w}{2}f'{\left(f''\right)}^{2}+Q\theta +\mathrm{Pr}EcKp{\left(f'\right)}^{2}+\mathrm{Pr}EcM{\left(f'\right)}^{2} \end{array}\right\}=0$$ (11)

$$\begin{array}{l} \phi ''+\left[f\phi '-\phi f'-\sigma_{m}\phi {\left(1+\delta \theta \right)}^{n}\exp\left(\frac{-E}{1+\delta \theta }\right)\right]Sc+\frac{Nt}{Nb}\theta ''=0\\ \phi ''+\left[f\phi '-\phi f'-A\phi {\left(1+\delta \theta \right)}^{n}\exp\left(\frac{-E}{1+\delta \theta }\right)\right]Sc+\frac{Nt}{Nb}\theta ''=0 \end{array}$$ (12)

$$\begin{array}{l} f'(0)=1,f(0)=-s,\phi (0)=1,\theta (0)=1,\chi (0)=1,as\eta =0\\ f'(\infty )\to 0,\phi (\infty )\to 0,\theta (\infty )\to 0,\chi (\infty )\to 0as\eta \to \infty \end{array}$$ (13)

The constraints reduce to


$$\begin{array}{l} f'(0)=1,f(0)=-s,\phi (0)=1,\theta (0)=1,\chi (0)=1,as\eta =0\\ f'(\infty )\to 0,\phi (\infty )\to 0,\theta (\infty )\to 0,\chi (\infty )\to 0as\eta \to \infty \end{array}$$ (14)

The corresponding structures appear in the study.

$M=\frac{2\sigma B_{0}^{2}l}{\rho U_{w}}$, $\lambda =\frac{\left(1-C_{\infty }\right)\beta g(T_{w}-T_{\infty })2l}{U_{w}^{2}}$, $Nr=\frac{\left(\rho_{p}-\rho_{f})(C_{w}-C_{\infty }\right)}{\beta (1-C_{\infty })\rho (T_{w}-T_{\infty })}$, $Rb=\frac{\left(\rho_{m}-\rho_{f})\gamma (N_{w}-N_{\infty }\right)}{\rho (1-C_{\infty })\beta (T_{w}-T_{\infty })}$

$Nt=\frac{\tau D_{T}(T_{w}-T_{\infty })}{\upsilon T_{\infty }}$, $Nb=\frac{\tau D_{B}(T_{w}-T_{\infty })}{\upsilon }$, $Z=\frac{k}{\mu }$, $\sigma_{1}=\frac{N_{\infty }}{N_{w}-N_{\infty }}$, $\sigma_{m}=\frac{2K_{r}^{2}l}{U_{w}}$, $E=\frac{E_{a}}{KT_{\infty }}$, $K=\frac{4\sigma^{*}T_{\infty }^{3}}{k^{*}K}$,

$Sc=\frac{\upsilon }{D}$, $Sb=\frac{\upsilon }{D_{n}}$, $Pe=\frac{dW_{c}}{D_{n}}$, $\delta =\frac{\left(T_{w}-T_{\infty }\right)}{T_{\infty }}$, $Ec=\frac{U_{w}^{2}}{C_{P}(T_{w}-T_{\infty })}$, $\mathrm{Pr}=\frac{\upsilon }{\alpha }$, $Q=\frac{Q_{0}}{u_{w}\rho C_{P}}$

Where M is the magnetic field stricture, Pr is the Prandtl number, W is the dimensionless Williamson liquid constraint, S < 0 is the suction constraint, S >  0 is the injection constraint, λ is the mixed convection constraint, Nr is the buoyancy ratio number, Rb is the bioconvection Rayleigh number, Nt *δ* is the thermophoresis constraint, Nb is the Brownian factor, $\sigma_{m}$ represents the dimensionless reaction rate. E denotes activation energy, K as the radiation stricture, $\sigma_{1}$ as the bioconvective difference constraint, *δ* is the temperature differentiation constraint, Sc as the Schmidt number, Sb as the Bioconvective Schmidt number, and Pe as the Peclet number, Ec is the Eckert number, Q is the heat generation.

The dimensionless strictures provided by


$$Cf_{x}=\frac{\tau_{w}}{\rho U_{w}^{2}},Nu_{x}=\frac{xq_{w}}{k(T_{w}-T_{\infty })},Sh_{x}=\frac{xq_{m}}{D_{B}(C_{w}-C_{\infty })},Nn_{x}=\frac{xq_{n}}{D_{N}(N_{w}-N_{\infty })},$$ (15)

Shear stress, heat flux, mass flux, and motile microbes are illustrations of $\tau_{w}$$q_{w}$ this, $q_{m}$$q_{n}$ respectively.


$$\tau_{w}=\mu {\left(\frac{\partial u}{\partial y}+\frac{\Gamma }{\sqrt{2}}{\left(\frac{\partial u}{\partial y}\right)}^{2}\right)}_{y=0},q_{w}=-{\left(K+\frac{4\sigma^{*}T_{\infty }^{3}}{k^{*}}\left(\frac{\partial T}{\partial y}\right)\right)}_{y=0},q_{m}=-D_{B}{\left(\frac{\partial C}{\partial y}\right)}_{y=0},q_{n}=-D_{N}{\left(\frac{\partial N}{\partial y}\right)}_{y=0}$$ (16)

We obtain these quantities by solving in terms of a given similarity transformation, we get:


RexCfx=f''(0)+λ2f''(0)2,NuxRex=−1+43Kθ'(0),ShxRex=−ϕ'(0),NnxRex=−χ'(0) (17)

Here, the local Reynolds number is Rex=xUw(x)υ.

## 3.  Analytical solution by homotopy analysis method

We utilize the HAM to explain Equations (10-13) below the given boundary restrictions (14). The results are obtained through a systematic process, and the inclusion of additional constraints enhances and stabilizes the concurrence of the outcomes.

The prime presumptions are designated as follows


$$f_{0}(\eta )=1+S-e^{-\eta },\theta_{0}(\eta )=e^{-\eta },\phi_{0}(\eta )=e^{-\eta },\chi_{0}=e^{-\eta }$$ (18)

The linear operatives are engaged as $L_{f},{\text{L}}_{\theta }{\text{,L}}_{\phi },{\text{L}}_{\chi }$:


$$L_{f}(f)=\frac{d^{3}f}{d\eta^{3}}-\frac{df}{d\eta },{\text{L}}_{\theta }\text{(}\theta \text{)=}\frac{d^{2}\theta }{d\eta^{2}}-\theta ,{\text{L}}_{\phi }\text{(}\phi \text{)=}\frac{d^{2}\phi }{d\eta^{2}}-\phi ,{\text{L}}_{\chi }\text{(}\chi \text{)=}\frac{d^{2}\chi }{d\eta^{2}}-\chi$$ (19)

Which have the resulting possessions:


$$L_{f}(c_{1}+c_{2}e^{-\eta }+c_{3}e^{\eta })=0,{\text{L}}_{\theta }(c_{4}e^{-\eta }+c_{5}e^{\eta })=0,{\text{L}}_{\phi }(c_{6}e^{-\eta }+c_{7}e^{\eta })=0,{\text{L}}_{\chi }(c_{8}e^{-\eta }+c_{9}e^{\eta })=0$$ (20)

Where $c_{i}(i=1-9)$ are the quantities in the general result:

The consequential non-linear operators $N_{f},N_{\theta }\text{,}N_{\phi },N_{\chi }$ are specified as follows:


$$\begin{array}{l} N_{f}\left[f(\eta ;p),\theta (\eta ;p),\phi (\eta ;p),\chi (\eta ;p)\right]=\frac{\partial^{3}f(\eta ;p)}{\partial \eta^{3}}-(M+K_{p})\frac{\partial f(\eta ;p)}{\partial \eta }-{\left(\frac{\partial f(\eta ;p)}{\partial \eta }\right)}^{2}+\\ f(\eta ;p)\frac{\partial^{2}f(\eta ;p)}{\partial \eta^{2}}+W\frac{\partial^{2}f(\eta ;p)}{\partial \eta^{2}}\frac{\partial^{3}f(\eta ;p)}{\partial \eta^{3}}+\lambda \left(\theta (\eta ;p)-Nr\phi (\eta ;p)-Rb\chi (\eta ;p)\right), \end{array}$$ (21)

$$\begin{array}{l} N_{\theta }\left[f(\eta ;p),\theta (\eta ;p),\phi (\eta ;p)\right]=\left(1+\frac{4}{3}K\right)\frac{\partial^{2}\theta (\eta ;p)}{\partial \eta^{2}}+\mathrm{Pr}Ec{\left(\frac{\partial^{2}f(\eta ;p)}{\partial \eta^{2}}\right)}^{2}+\\ \mathrm{Pr}\left(f(\eta ;p)\frac{\partial \theta (\eta ;p)}{\partial \eta }-\frac{\partial f(\eta ;p)}{\partial \eta }\theta (\eta ;p)+Nb\frac{\partial \theta (\eta ;p)}{\partial \eta }\frac{\partial \phi (\eta ;p)}{\partial \eta }+Nt{\left(\frac{\partial \theta (\eta ;p)}{\partial \eta }\right)}^{2}\right)+\\ \mathrm{Pr}EcM{\left(\frac{\partial f(\eta ;p)}{\partial \eta }\right)}^{2}+\mathrm{Pr}EcKp{\left(\frac{\partial f(\eta ;p)}{\partial \eta }\right)}^{2}+Q\theta (\eta ;p)+\mathrm{Pr}\frac{w}{2}\frac{\partial f(\eta ;p)}{\partial \eta }{\left(\frac{\partial^{2}f(\eta ;p)}{\partial \eta^{2}}\right)}^{2}, \end{array}$$ (22)

$$\begin{array}{l} N_{\phi }\left[f(\eta ;p),\theta (\eta ;p),\phi \left(\eta ;p\right)\right]=\frac{\partial^{2}\phi (\eta ;p)}{\partial \eta^{2}}+\left(\frac{Nt}{Nb}\right)\frac{\partial^{2}\theta \left(\eta ;p\right)}{\partial \eta^{2}}+\\ Sc\left(\begin{array}{l} f(\eta ;p)\frac{\partial \phi \left(\eta ;p\right)}{\partial \eta }-\frac{\partial f(\eta ;p)}{\partial \eta }\phi \left(\eta ;p\right)-\\ \sigma_{m}\phi (\eta ;p)(1+\delta \theta {\left(\eta ;p\right)}^{m}\exp\left(\frac{-E}{1+\delta \theta (\eta ;p)}\right) \end{array}\right) \end{array}$$ (23)

$$\begin{array}{l} N_{\chi }\left[f(\eta ;p),\phi (\eta ;p),\chi (\eta ;p)\right]=\frac{\partial^{2}\chi (\eta ;p)}{\partial \eta^{2}}+\chi (\eta ;p)\frac{\partial^{2}\phi (\eta ;p)}{\partial \eta^{2}}+\frac{\partial \phi (\eta ;p)}{\partial \eta }\frac{\partial \chi (\eta ;p)}{\partial \eta }\\ Sb\mathrm{Pr}\left(f(\eta ;p)\frac{\partial \chi (\eta ;p)}{\partial \eta }-\frac{\partial f(\eta ;p)}{\partial \eta }\chi (\eta ;p)\right)-Pe\Omega \frac{\partial^{2}\phi (\eta ;p)}{\partial \eta^{2}} \end{array}$$ (24)

The elementary knowledge of HAM is designated in [2–5], the zero^th^-order problems from Equations. (10-13) are:


$$(1-p)L_{f}\left[f(\eta ;p)-f_{0}(\eta )\right]=p\hbar_{f}N_{f}\left[f(\eta ;p),\theta (\eta ;p),\phi (\eta ;p),\chi (\eta ;p)\right]$$ (25)

$$(1-p)L_{\theta }\left[\theta (\eta ;p)-\theta_{0}(\eta )\right]=p\hbar_{\theta }N_{\theta }\left[f(\eta ;p),\theta (\eta ;p),\phi (\eta ;p)\right]$$ (26)

$$(1-p)L_{\phi }\left[\phi (\eta ;p)-\phi_{0}(\eta )\right]=p\hbar_{\phi }N_{\phi }\left[f(\eta ;p),\theta (\eta ;p),\phi (\eta ;p)\right]$$ (27)

$$(1-p)L_{\chi }\left[\chi (\eta ;p)-\chi_{0}(\eta )\right]=p\hbar_{\chi }N_{\chi }\left[f(\eta ;p),\phi (\eta ;p),\chi (\eta ;p)\right]$$ (28)

The equivalent boundary restrictions are:


$$\begin{array}{l} {\left.f(\eta ;p)\right|}_{\eta =0}=S,{\left.\frac{df(\eta ;p)}{d\eta }\right|}_{\eta =0}=1,{\left.\theta (\eta ;p)\right|}_{\eta =0}=1,\\ {\left.\phi (\eta ;p)\right|}_{\eta =0}=1,{\left.\chi (\eta ;p)\right|}_{\eta =0}=1,{\left.\frac{df(\eta ;p)}{d\eta }\right|}_{\eta \to \infty }=0,\\ {\left.\theta (\eta ;p)\right|}_{\eta \to \infty }=0,{\left.\phi (\eta ;p)\right|}_{\eta \to \infty }=0,{\left.\chi (\eta ;p)\right|}_{\eta \to \infty }=0, \end{array}$$ (29)

Where p∈[0,1] the entrenching stricture $\hbar_{f},\hbar_{\theta }\text{, }\hbar_{\phi },\hbar_{\chi }$ is used to resist the convergence of the result. When p=0and p=1 we have:


f(η;1)=f(η),θ(η;1)=θ(η),ϕ(η;1)=ϕ(η),χ(η;1)=χ(η), (30)

Intensifying f(η;p),θ(η;p),ϕ(η;p),χ(η;p), in Taylor’s series about p=0


$$\begin{array}{l} f(\eta ;p)=f_{0}(\eta )+\sum_{m=1}^{\infty }f_{m}(\eta )p^{m},\theta (\eta ;p)=\theta_{0}(\eta )+\sum_{m=1}^{\infty }\theta_{m}(\eta )p^{m},\\ \phi (\eta ;p)=\phi_{0}(\eta )+\sum_{m=1}^{\infty }\phi_{m}(\eta )p^{m},\chi (\eta ;p)=\chi_{0}(\eta )+\sum_{m=1}^{\infty }\chi_{m}(\eta )p^{m}, \end{array}$$ (31)

Where


$$\begin{array}{l} f_{m}(\eta )={\left.\frac{1}{m!}\frac{\partial f(\eta ;p)}{\partial \eta }\right|}_{p=0},\theta_{m}(\eta )={\left.\frac{1}{m!}\frac{\partial \theta (\eta ;p)}{\partial \eta }\right|}_{p=0}\text{,}\\ \phi_{m}(\eta )={\left.\frac{1}{m!}\frac{\partial \phi (\eta ;p)}{\partial \eta }\right|}_{p=0},\chi_{m}(\eta )={\left.\frac{1}{m!}\frac{\partial \chi (\eta ;p)}{\partial \eta }\right|}_{p=0} \end{array}$$ (32)

The subordinate restrictions are chosen to ensure that the series converges (29), resulting in the acquisition of the expression found in (25):


$$\begin{array}{l} f(\eta )=f_{0}(\eta )+\sum_{m=1}^{\infty }f_{m}(\eta ),\theta (\eta )=\theta_{0}(\eta )+\sum_{m=1}^{\infty }\theta_{m}(\eta ),\\ \phi (\eta )=\phi_{0}(\eta )+\sum_{m=1}^{\infty }\phi_{m}(\eta ),\chi (\eta )=\chi_{0}(\eta )+\sum_{m=1}^{\infty }\chi_{m}(\eta ), \end{array}$$ (33)

The *m* th-order delinquent gratifies the resulting:


$$\begin{array}{l} L_{f}\left[f_{m}(\eta )-\omega_{m}f_{m-1}(\eta )\right]=\hbar_{f}R_{m}^{f}(\eta ),L_{\theta }\left[\theta_{m}(\eta )-\omega_{m}\theta_{m-1}(\eta )\right]=\hbar_{\theta }R_{m}^{\theta }(\eta ),\\ L_{\phi }\left[\phi_{m}(\eta )-\omega_{m}\phi_{m-1}(\eta )\right]=\hbar_{\phi }R_{m}^{\phi }(\eta ),L_{\chi }\left[\chi_{m}(\eta )-\omega_{m}\chi_{m-1}(\eta )\right]=\hbar_{\chi }R_{m}^{\chi }(\eta ), \end{array}$$ (34)

The consistent boundary restrictions are:


$$\begin{array}{l} f_{m}(0)={f^{\prime }}_{m}(0)=\theta_{m}(0)=\phi_{m}(0)=\chi_{m}(0)=0\\ {f^{\prime }}_{m}(\infty )=\theta_{m}(\infty )=\phi_{m}(\infty )=\chi_{m}(\infty )=0 \end{array}$$ (35)

Here


$$\begin{array}{l} R_{m}^{f}(\eta )={f^{\prime \prime \prime }}_{m-1}-(M+K_{p}){f^{\prime }}_{m-1}+\sum_{k=0}^{m-1}f_{m-1-k}{f^{\prime \prime }}_{k}-2\sum_{k=0}^{m-1}{f^{\prime }}_{m-1-k}{f^{\prime }}_{k}-\\ W\sum_{k=0}^{m-1}{f^{\prime \prime \prime }}_{m-1-k}{f^{\prime \prime }}_{k}+\lambda \left(\theta_{m-1}-Nr\phi_{m-1}-Rb\chi_{m-1}\right), \end{array}$$ (36)

$$\begin{array}{l} R_{m}^{\theta }(\eta )=\left(1+\frac{4}{3}K\right){\theta^{\prime \prime }}_{m-1}+\mathrm{Pr}Ec\sum_{k=0}^{m-1}{f^{\prime \prime }}_{m-1-k}{f^{\prime \prime }}_{k}+\mathrm{Pr}Ec(M+Kp)\sum_{k=0}^{m-1}{f^{\prime }}_{m-1-k}{f^{\prime }}_{k}+\\ \mathrm{Pr}\left(\sum_{k=0}^{m-1}f_{m-1-k}{\theta^{\prime }}_{k}-\sum_{k=0}^{m-1}{f^{\prime }}_{m-1-k}\theta_{k}+Nb\sum_{k=0}^{m-1}{\theta^{\prime }}_{m-1-k}{\phi^{\prime }}_{k}+Nt\sum_{k=0}^{m-1}{\theta^{\prime }}_{m-1-k}{\theta^{\prime }}_{k}\right)+Q\theta_{m-1}+\\ \mathrm{Pr}\frac{W}{2}\sum_{k=0}^{m-1}{f^{\prime }}_{m-1-k}\sum_{l=0}^{k}{f^{\prime \prime }}_{k-l}{f^{\prime \prime }}_{l}, \end{array}$$ (37)

$$\begin{array}{l} R_{m}^{\phi }(\eta )={\phi^{\prime \prime }}_{m-1}+\left(\frac{Nt}{Nb}\right){\theta^{\prime \prime }}_{m-1}+\\ Sc\left(\sum_{k=0}^{m-1}f_{m-1-k}{\phi^{\prime }}_{k}-\sum_{k=0}^{m-1}{f^{\prime }}_{m-1-k}\phi_{k}-\sigma_{m}\phi_{m-1}{\left(1+\delta \theta_{m-1}\right)}^{m}\exp\left(\frac{-E}{1+\delta \theta_{m-1}}\right)\right), \end{array}$$ (38)

$$\begin{array}{l} R_{m}^{\chi }(\eta )={\chi^{\prime \prime }}_{m-1}+Sb\mathrm{Pr}\left(\sum_{k=0}^{m-1}f_{m-1-k}{\chi^{\prime }}_{k}-\sum_{k=0}^{m-1}{f^{\prime }}_{m-1-k}\chi_{k}\right)\\ -Pe\Omega {\phi^{\prime \prime }}_{m-1}\text{+}\sum_{k=0}^{m-1}{\phi^{\prime \prime }}_{m-1-k}\chi_{k}\sum_{k=0}^{m-1}{\phi^{\prime }}_{m-1-k}{\chi^{\prime }}_{k}, \end{array}$$ (39)

where


$$\omega_{m}= \{\begin{array}{l} 0,\text{if p}\leq \text{1}\\ 1,\text{if p}>\text{1} \end{array}$$

The following are the general solutions:


$$\begin{array}{l} L_{f}(\eta )=f_{m}^{*}(\eta )+c_{1}+c_{2}e^{-\eta }+c_{3}e^{\eta }\\ \theta_{m}(\eta )=\theta_{m}^{*}(\eta )+c_{4}e^{-\eta }+c_{5}e^{\eta }\\ \phi_{m}(\eta )=\phi_{m}^{*}(\eta )+c_{6}e^{-\eta }+c_{7}e^{\eta }\\ \chi_{m}(\eta )=\chi_{m}^{*}(\eta )+c_{8}e^{-\eta }+c_{9}e^{\eta } \end{array}$$ (40)

then $f_{m}^{*}(\eta ),\theta_{m}^{*}(\eta ),\phi_{m}^{*}(\eta ),\chi_{m}^{*}(\eta )$ are the particular solutions. MATHEMATICA is employed to address linear homogeneous equations (33) and (34) in a consecutive manner of m =  1,2,3.

### Convergence of HAM solution

**Fig 2:**
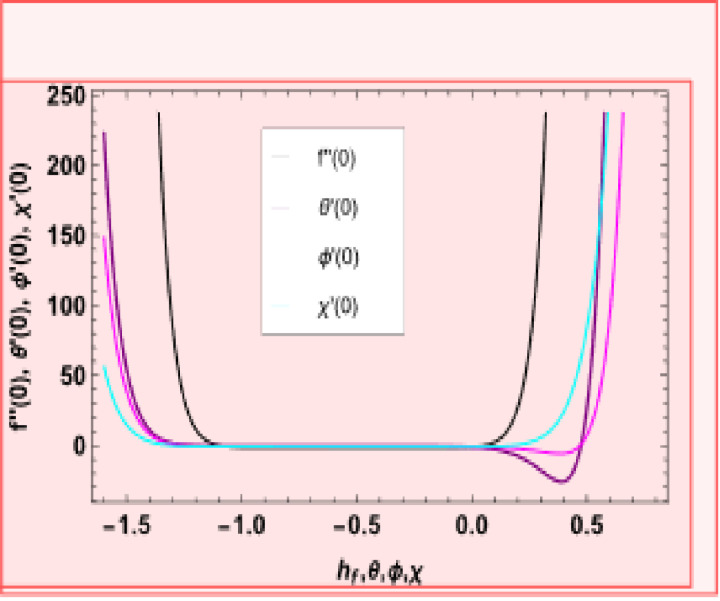
h-curve for function f''(0),θ'(0),ϕ'(0),χ'(0).

The equations employ series expansions to obtain solutions. Fig 2 illustrates the h-curves at the 18th order of approximation, assisting in determining suitable values for $h_{f},h_{\theta },h_{\phi }$ and $h_{\chi }$. Fig 2 presents the suitable values of ℏ is $-1.75\leq h_{f}\leq -0.4,$, $-1.82\leq h_{\theta }\leq -0.28$, $-1.73\leq h_{\phi }\leq -0.38$, and $-1.73\leq h_{\chi }\leq -0.38$. Our calculations signify that the solutions series meets in the whole region of *η* when $h_{f}=-1.50572$, $h_{\theta }=-0.9$, $h_{\phi }=-0.857873$, and $h_{\chi }=-0.876543$. Table 1 was designed solely to define the orders of approximation required.

**Table 1:** HAM solutions convergence at various approximation order.

m	f''(0)	θ'(0)	ϕ'(0)	χ'(0)
4	−1.2067	−0.791399	−0.862374	−0.900977
8	−1.30045	−0.708235	−0.832171	−0.895081
12	−1.34442	−0.643715	−0.808086	−0.885713
16	−1.36533	−0.592977	−0.788852	−0.874855
20	−1.37542	−0.552520	−0.77343	−0.863655
24	−1.38056	−0.51984	−0.760888	−0.852753
28	−1.38316	−0.492979	−0.750650	−0.84247
32	−1.38453	−0.5092654	−0.748953	−0.83798
36	−1.38522	−0.5083241	−0.748645	−0.83689
38	−1.38547	−0.5082352	−0.748593	−0.83634

### Convergent table

The Homotopy Analysis Method is used to solve non-linear ordinary differential equations (ODEs) and achieve precise solutions. This analytical technique allows for selecting suitable base functions to approximate non-linear problems, ensuring the straightforward convergence of the solution series. Here N[u(x)]=0, represents a non-linear operator, defining the equation is $\left(1-q)[(x;q)-u_{0}(x;q)]=h_{q}N[U(x;q)\right]$. Consider $u_{0}(x)$ the initial estimation for the eventual solution *u*(*x*), where ℏ is a constant.

**Fig 3:**
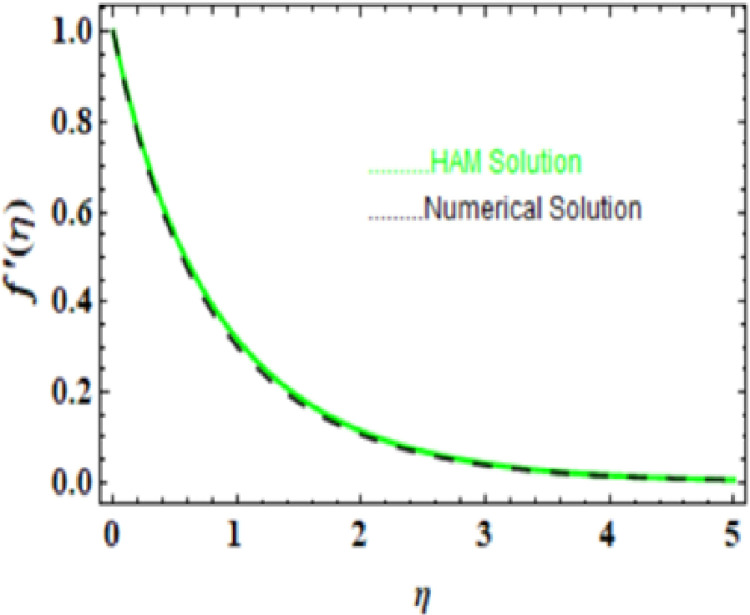
HAM and Numerical solution for f'(η).

## 4.  Results and discussion

Table 2. Comparison of skin friction for different values of *λ* and M. Table 3 displays the f''(0) record, which upsurges M, Rb, Nr and Kp but declines for λ. Table 4 demonstrates that it progresses with Pr but diminutions with K, Kp, Ec, Nb, and Nt. Table 5 illustrates that retreats only E, A upsurges, whereas improvements directly with Sc, $\sigma_{m}$. Finally, table 6 demonstrates that the motile density number increases directly to Sb, *Ω* and Pe.

**Table 2:** Comparison of $\sqrt{Re_{x}}Cf$ with changed values of *λ* and M.

*λ*	M	Asjad et al [28]	Present result
0.1	2.0	1.754213	1.729181
0.2		1.678675	1.631944
0.3		1.579827	1.559550
	0.1	1.201556	1.189670
	0.2	1.237223	1.235811
	0.3	1.271816	1.253232

**Table 3:** The significance of the specific Nusselt number on a variety of factors.

M	Kp	λ	Nr	Rb	– f''(0)
0.5					0.6850
1.0					0.6943
1.5					0.7018
	0.1				0.6764
	0.4				0.6830
	0.7				0.6889
		0.2			1.0097
		0.5			0.8097
		0.8			0.6177
			0.1		1.0764
			0.5		1.0798
			0.9		1.0834
				0.1	1.0764
				0.5	1.0798
				1.0	1.0843

**Table 4:** The significance of the specific Nusselt number on a variety of factors.

Pr	Q	Ec	Nb	Nt	−θ'(0
0.1					0.9734
0.2					0.9760
0.3					0.9785
	0.1				1.0261
	0.5				0.9814
	1.0				0.9247
		0.2			1.0106
		0.5			0.9908
		0.8			0.9709
			0.1		0.9930
			0.3		0.9709
			0.5		0.9488
				0.1	0.9930
				0.4	0.9598
				0.7	0.9267

**Table 5:** The significance of the specific Nusselt number on a variety of factors.

$\sigma_{m}$	E	Sc	$-\Phi \text{'}\left(0\right)$
2.0			0.8825
3.0			0.9103
4.0			0.9380
	0.2		0.8353
	0.4		0.8344
	0.6		0.8326
		0.2	0.8236
		0.5	0.8589
		0.8	0.8942

**Table 6:** The significance of the specific Nusselt number on a variety of factor.

Sb	Pe	*Ω*	$-\text{χ '}\left(0\right)$
0.1			0.9657
0.4			1.0008
0.7			1.0359
	0.1		0.9531
	0.3		0.0891
	0.5	0.2	1.0251
		0.5	0.9861
		0.8	0.9951
			1.0041

### Validation

**Fig 4:**
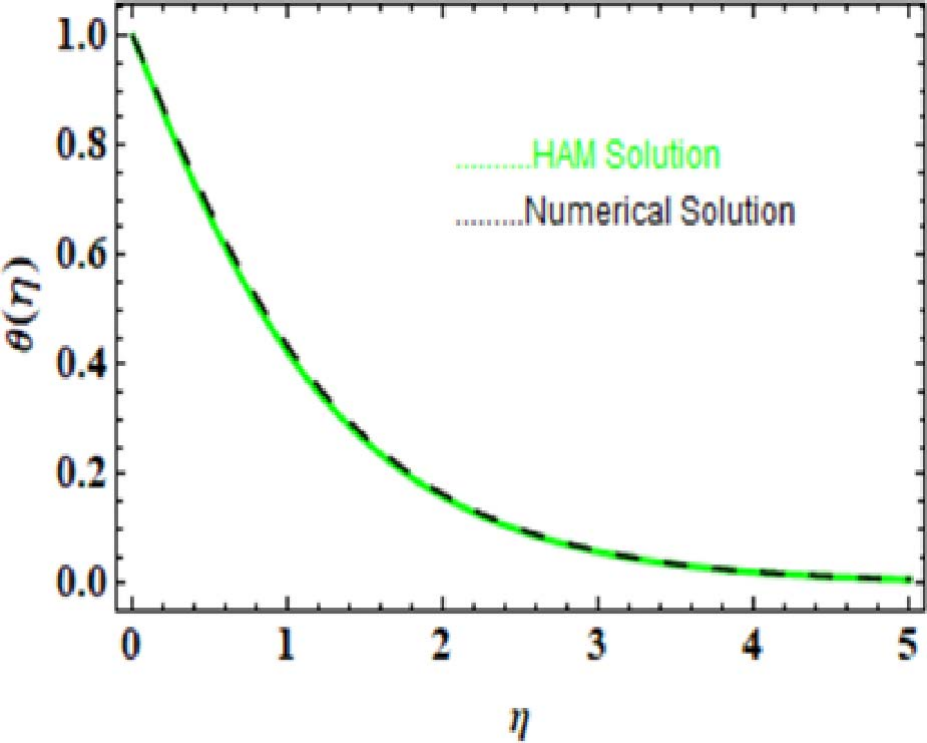
HAM and Numerical solution for θ(η).

**Fig 5:**
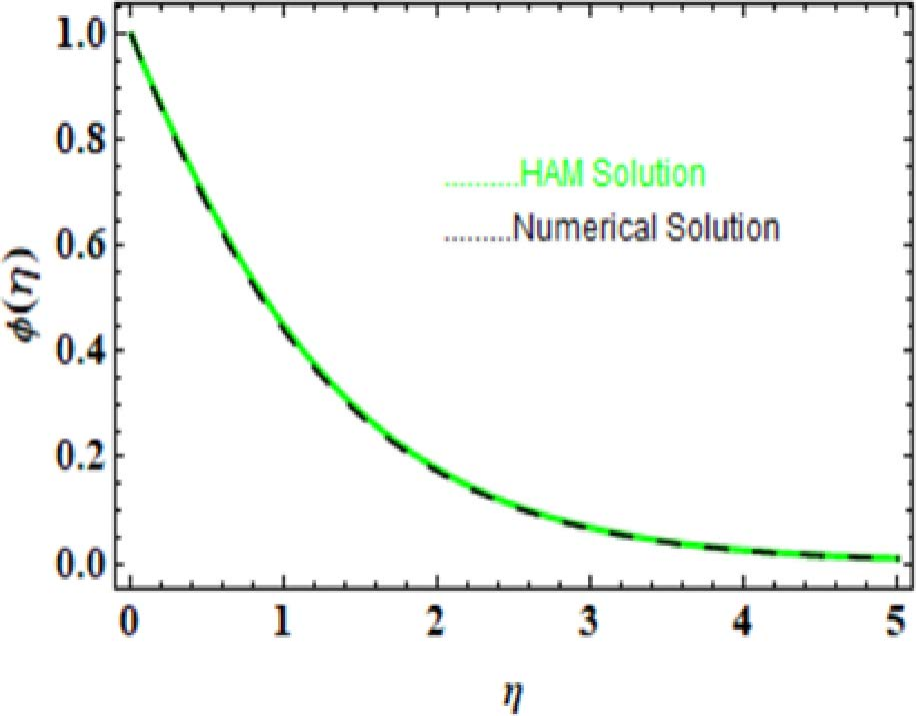
HAM and Numerical solution for ϕ(η).

**Fig 6:**
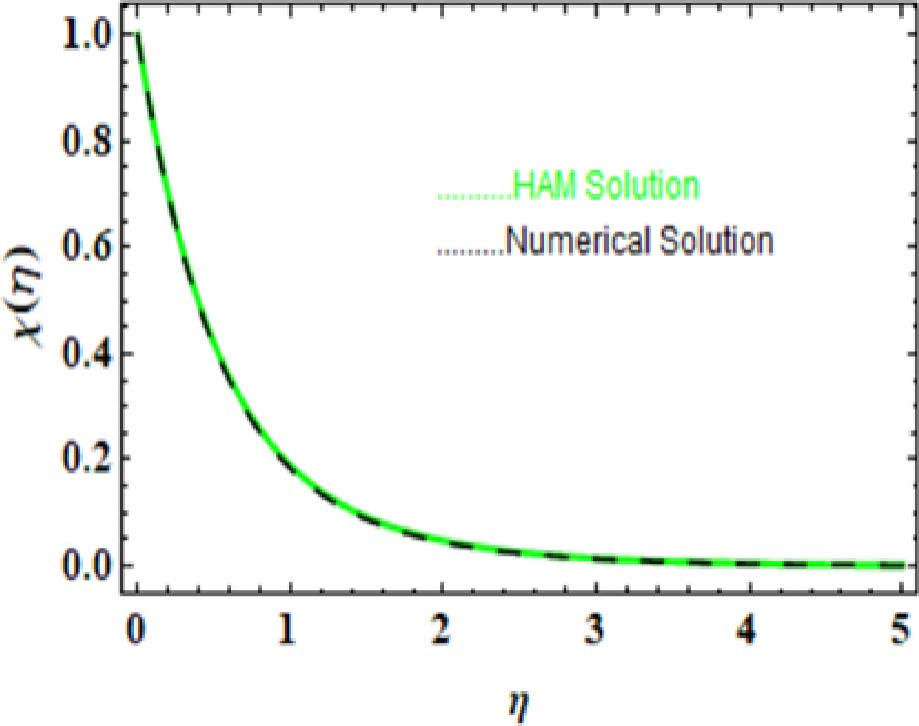
HAM and Numerical solution for χ(η).

we compared the numerical solutions for velocity, temperature, and concentration, as shown in Figs [3-6], with the HAM solutions. The comparisons showed good agreement. Furthermore, we validated the accuracy of our method by comparing our numerical results with the HAM solutions in Tables 7-10, which also confirmed the reliability of our approach.

**Table 7:** HAM and Numerical solution for f'(η).

*η*	HAM solution	Numerical soltuion	Absolute Error
0.0	1.000000	1.000000	0.000000
0.5	0.547551	0.547004	0.001095
1.0	0.317109	0.316574	0.001068
1.5	0.188260	0.187860	0.000800
2.0	0.113008	0.112737	0.000543
2.5	0.068183	0.068007	0.000351
3.0	0.041239	0.041128	0.000221
3.5	0.024974	0.024905	0.000137
4.0	0.015134	0.015092	0.000084
4.5	0.009174	0.009149	0.000051
5.0	0.005563	0.005547	0.000031

**Table 8:** HAM and Numerical solution for θ(η).

*η*	HAM solution	Numerical soltuion	Absolute Error
0.0	1.000000	1.000000	0.000000
0.5	0.667258	0.667732	0.000949
1.0	0.421523	0.421927	0.000809
1.5	0.259895	0.260168	0.000545
2.0	0.158662	0.158833	0.000343
2.5	0.096482	0.096588	0.000211
3.0	0.058581	0.058645	0.000128
3.5	0.035547	0.035586	0.000078
4.0	0.021564	0.021588	0.000047
4.5	0.013081	0.013095	0.000029
5.0	0.007934	0.007943	0.000017

**Table 9:** HAM and Numerical solution for ϕ(η).

η	HAM solution	Numerical soltuion	Absolute Error
0.0	1.000000	1.000000	0.000000
0.5	0.684080	0.683620	0.000460
1.0	0.448498	0.285023	0.000467
1.5	0.285380	0.285023	0.000357
2.0	0.178204	0.177960	0.000245
2.5	0.110009	0.109850	0.000159
3.0	0.067440	0.067339	0.000101
3.5	0.041170	0.041107	0.000062
4.0	0.025069	0.025030	0.000038
4.5	0.015241	0.015218	0.000024
5.0	0.009258	0.009243	0.000014

**Table 10:** HAM and Numerical solution for χ(η).

η	HAM solution	Numerical soltuion	Absolute Error
0.0	1.000000	1.000000	0.000000
0.5	0.419277	0.418959	0.000319
1.0	0.189241	0.188943	0.000298
1.5	0.092214	0.092000	0.000214
2.0	0.048006	0.047865	0.000141
2.5	0.026295	0.026206	0.000089
3.0	0.014932	0.014877	0.000055
3.5	0.008688	0.008654	0.000034
4.0	0.005135	0.005114	0.000021
4.5	0.003065	0.003053	0.000013
5.0	0.001841	0.001834	$7.71\times 10^{-6}$

**Fig 7:**
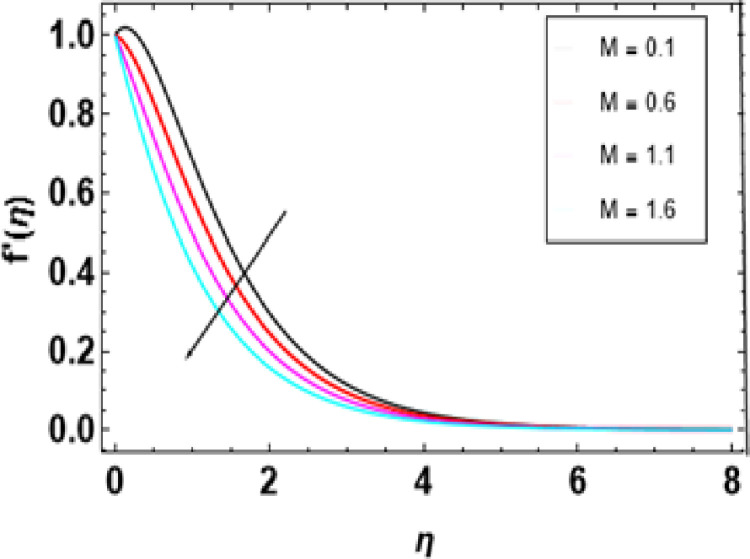
Outcomes of M on f'(η).

**Fig 8:**
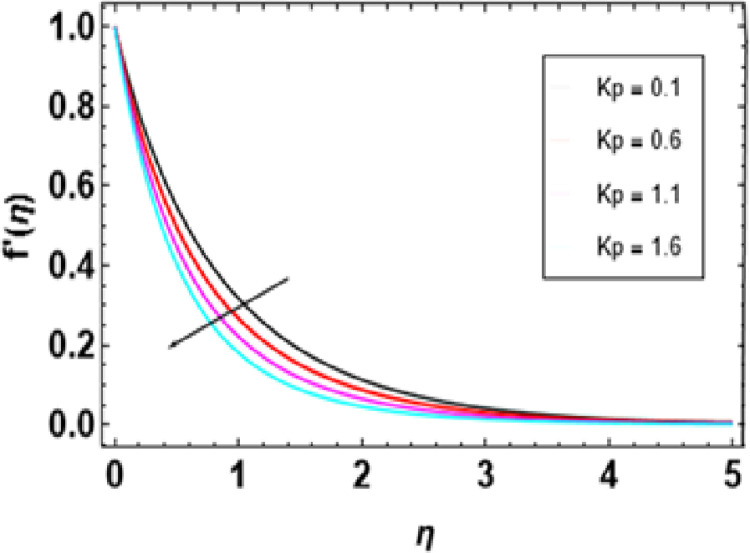
Outcomes of K on f'(η).

**Fig 9:**
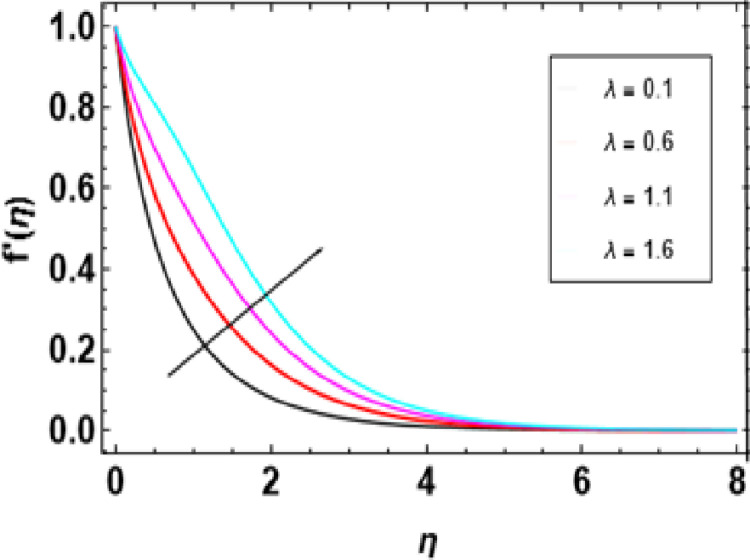
Outcomes of λ on f'(η).

**Fig 10:**
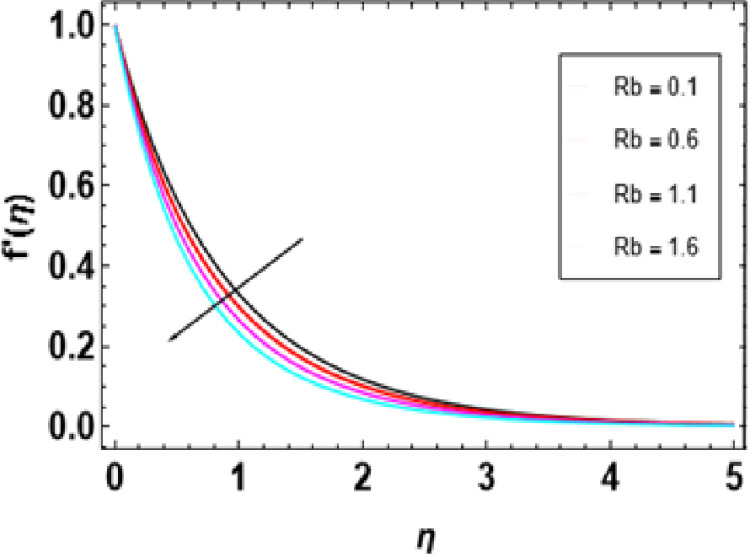
Outcomes of Rb on f'(η).

**Fig 11:**
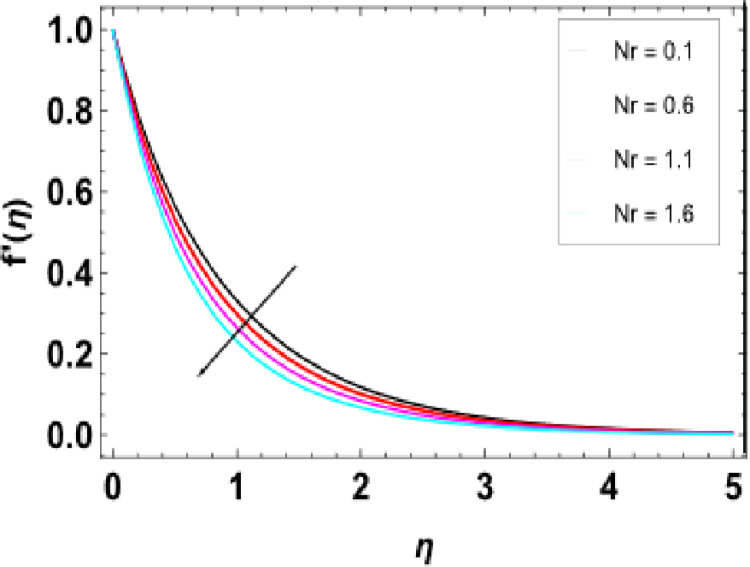
Outcomes of Nr on f'(η).

Fig 7 illustrates how this magnetic constraint on the velocity field is inspired. It can be seen that for any increase in M, fluid velocity decreases. This is related to Lorentz force that manifests due to the interaction between fluid and magnetic field motion. As M increases, fluid particles come closer together, increasing resistive force. The resistance force opposes the flow of fluid, known as Lorentz force, and causes expansion of the heat boundary layer, slowing down the flow by increasing the thickness. Thus, increased resistance leads to a decrease in the velocity of flow due to a thermal boundary layer being formed with its expansion, causing a rise in thickness. The effects of porosity parameters on velocity profiles are shown in Fig 8. The velocity field declines with increasing porosity parameters. Higher porosity or lower permeability introduces more hurdles within the porous structure, trapping the fluid within it, making it difficult to move through. As a result, the imposed force or pressure gradient becomes less effective in overcoming this resistance, lowering fluid velocity. The deceleration arises from higher porosity parameters, leading to increased resistance within the porous medium, thereby hindering the flow of nanofluid. Fig 9 reveals that, λ being a variable of mixed convection, the growing values of its function cause the velocity gradient f’(η). Mixed convection coefficient is a measure of the balance between buoyancy and viscous forces. The greater the parameter value on mixed convection, the more significant becomes a change in velocity due to domination of buoyancy over viscous flow effects. Thus, there is enhanced correlativity between buoyant and inertial forces so that velocity f’(η) increases in magnitude and also broadens the boundary layer. Fig 10 depicts the velocity field as a function of bioconvection Rayleigh number Rb. The flow velocity profile shows an apparent decrease as the bioconvection Rayleigh number Rb increases. This implies that larger values of these quantities bring about lesser fluid velocities and, therefore, slow motion within the system due to the combined effects of buoyancy forces and bioconvection. Fig 11 illustrates the velocity field and its relationship to the buoyancy ratio parameter Nr. As can be seen from Fig 10, fluid velocity decreases as the buoyancy ratio parameter increases. Higher values of Nr cause intensified buoyancy forces, thus resulting in a significant increase in fluid density near the surface. The increment in buoyancy is mainly due to bioconvection sources and it is responsible for reducing fluid velocity.

**Fig 12:**
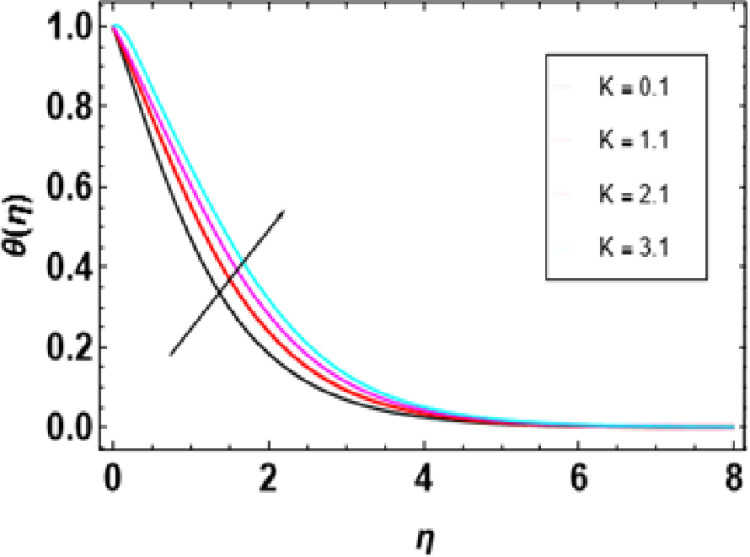
Outcomes of K on **θ**(η).

**Fig 13:**
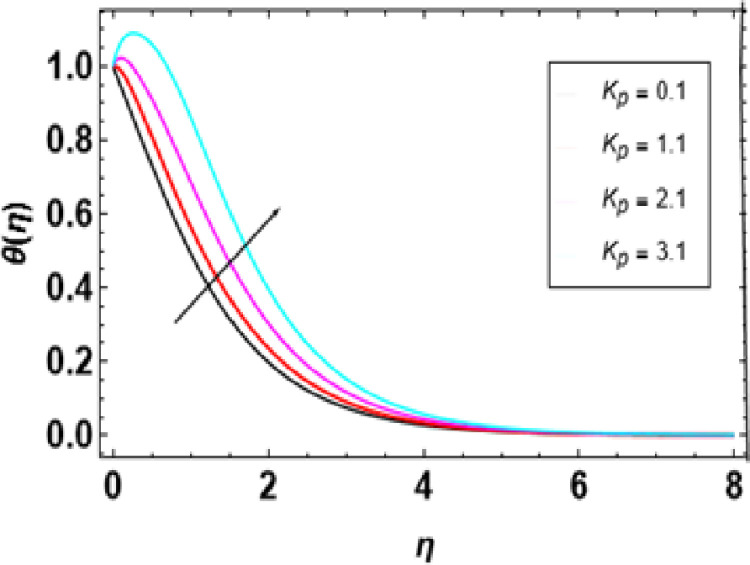
Outcomes of Kp on **θ**(η).

According to Fig 12, the radiation parameter affects temperature. Temperature profile increases with higher radiation parameters, as seen from a closer examination of the figure. This is because when there is a high radiation parameter, more heat comes into the fluid, leading to an increase in thermal boundary layer thickness and temperature. Thermal radiation enhances convective flow in such a way that the radiation parameter increases while flow velocity rises. Therefore, heat transfer is improved by thermal radiation while increasing the thickness of the thermal boundary layer as this parameter gets larger. The higher values of this parameter provide more heat for fluid so that both temperature and thermal boundary layer thickness become greater thus intensifying distribution of temperatures through it due to increased presence of heat in large quantities in it. Fig 13 depicts the influence of different porosity parameters on the temperature distributions in nanofluid flow. The graphical representations demonstrate how different properties change due to alterations in porosity parameters. It is also understood that the temperature distribution is also increased with an increasing value for porosity. A more significant porous parameter suggests highly compacted media that cause severe hindrance to the movement of nanoparticles in the nanofluid. Hence, when the porous parameter increases, the temperature of the nanofluid has to increase accordingly.

**Fig 14:**
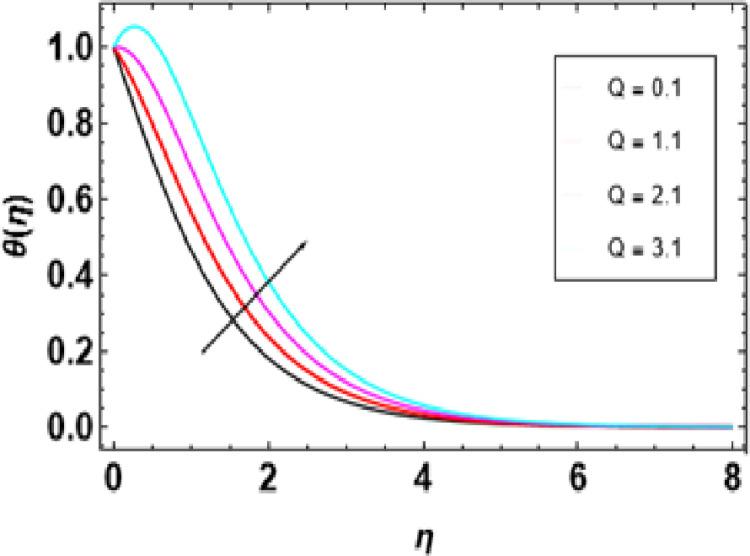
Outcomes of Q on **θ**(η).

**Fig 15:**
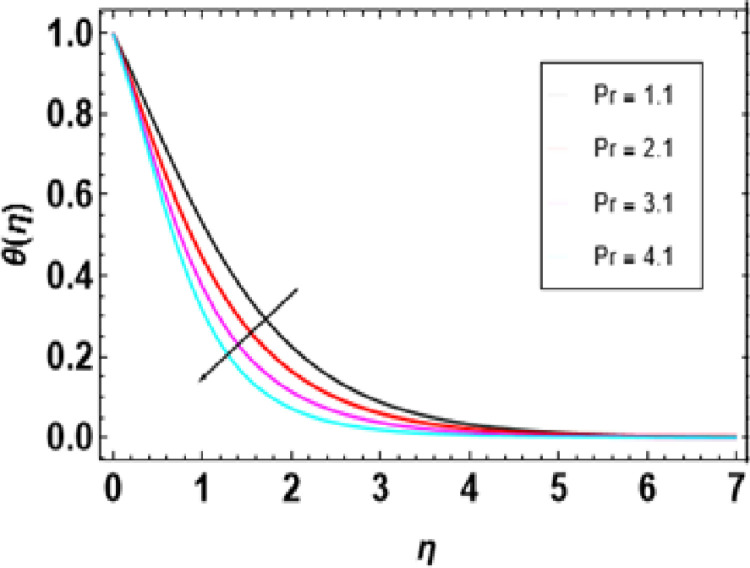
Outcomes of Pr on **θ**(η).

**Fig 16:**
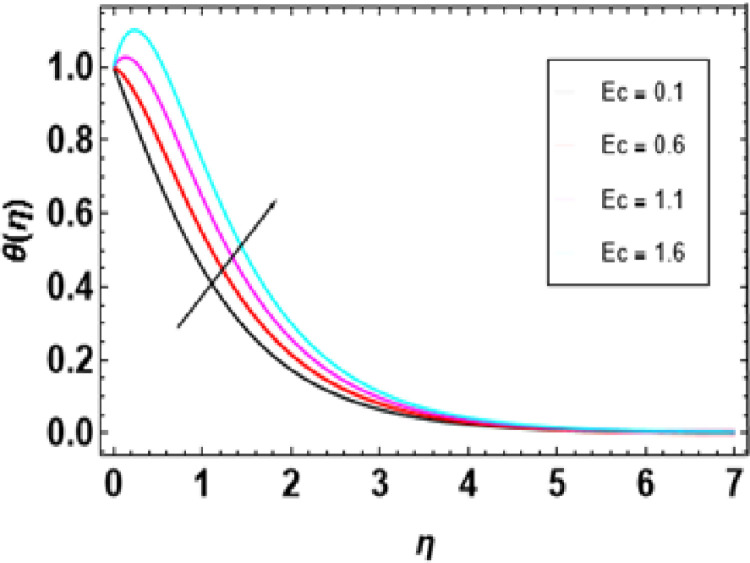
Outcomes of Ec on **θ**(η).

**Fig 17:**
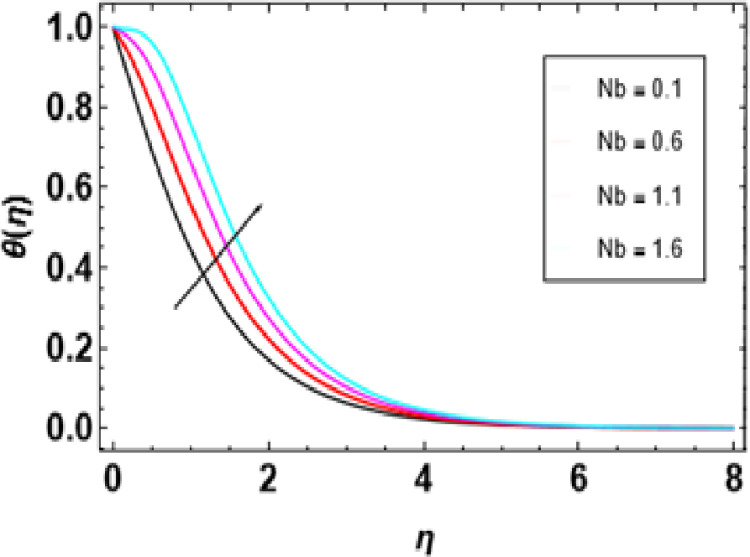
Outcomes of Nb on **θ**(η).

**Fig 18:**
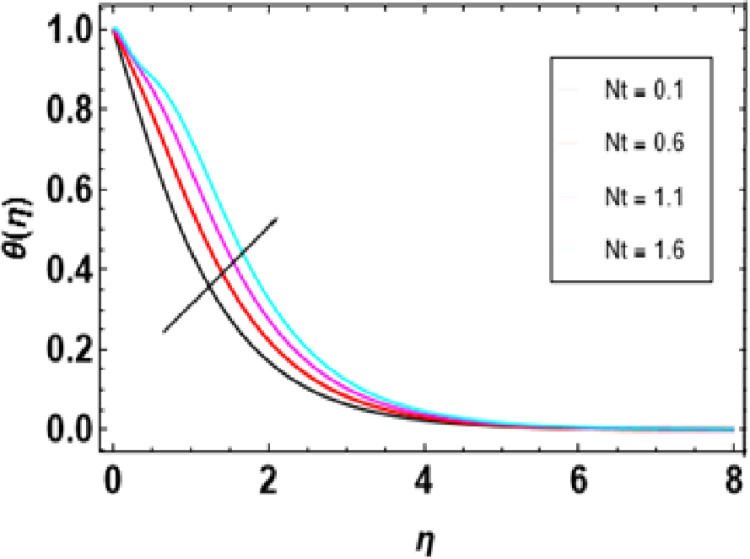
Outcomes of Nt on **θ**(η).

Heat generation adds thermal energy to the nanofluid, intensifying the vibrations of both the nanoparticles and the base fluid, leading to a rise in temperature. This process is crucial in determining the temperature profile of the nanofluid, with its effectiveness dependent on the heat source’s strength, the type of nanoparticles, and the flow conditions. Fig 14 demonstrates that an increase in the heat generation parameter leads to a higher temperature in the nanofluid. Engineers must consider the interaction between flow conditions, heat source strength, and nanoparticle properties when designing heat transfer systems to maintain the desired temperature range. Fig 15 represents the influence of the Prandtl number on temperature. As the Prandtl number increases, the temperature decreases. Higher Pr values decrease thermal diffusivity resulting in a thinner thermal boundary layer. When Pr <<1, it means that heat conduction is more significant than convection, which is dominated by a small value of the Prandtl number. For example, liquid mercury stands out in heat conduction due to its high thermal diffusivity. Conversely, there are fluids with large Pr numbers (Pr ≫ 1), like engine oil, where momentum diffusivity is dominant as fluid viscosity is high and heat conductivity tends to be low. The Prandtl number’s typical value for gases is around 1, meaning that momentum and heat dissipate at almost similar rates. In liquid metals (Pr <<1), heat diffuses far more quickly than momentum, resulting in a much thicker thermal boundary layer than the velocity boundary layer, as shown below. In contrast, oils(Pr ≫  1); have very thin thermal boundary layers because at this point, it takes a long time for heat to diffuse through them just like that It is important to note how the Prandtl number controls the relative thicknesses of these two boundaries since it plays an important role in solving problems associated with the transfer of energy. For low Prandtl numbers, heat diffusion is very fast compared to momentum or rate of fluid flow. This means the thermal boundary layer will be thicker, especially in liquid metals. As shown in Fig 16, we can observe the relationship between Eckert number and thermal energy. This graph demonstrates how a combined effect of Eckert numbers affects temperature profiles. The graph presents that the temperature and sheet temperatures increase with increasing Eckert. In terms of physics, this increased temperature with an increase in Eckert number implies that the system has a higher capacity to absorb or hold on to heat energy, implying enhanced heat adsorption properties. Fig 17 demonstrates the relationship between the Brownian motion constraint and the temperature field. Inside a liquid, particles have random motions, referred to as Brownian motion. This heat becomes very intense when Brownian movement increases, thus increasing this random motion. Also, the fluid’s temperature and the thickness of its thermal boundary layer increase simultaneously. Fundamentally, liquid molecules move more randomly with increased Brownian motion parameters. There is reduced molecular activity, hence lessening the thermal boundary layer and reducing general temperatures within the domain. The thermophoresis parameter in Fig 18 is increased, and consequently, the temperature profiles reduce gradually as the nanofluid particles shift to colder regions. This is due to enhanced heat transfer between the particles and surrounding fluid as they move towards cold regions. Hence, this causes a drop in their temperature since the colder fluid absorbs more thermal energy from them. More or less, it may be concluded that thermophoretic force, which acts more strongly for this case of study, pushes these particles into lower temperature areas where they come into contact with more excellent environments, leading to a reduction in their temperature by conduction to these surroundings.

**Fig 19:**
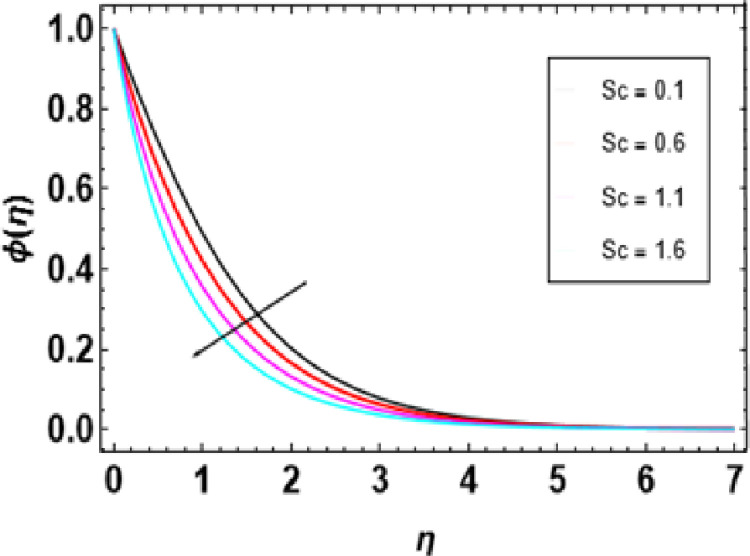
Outcomes of Sc onϕ(η).

**Fig 20:**
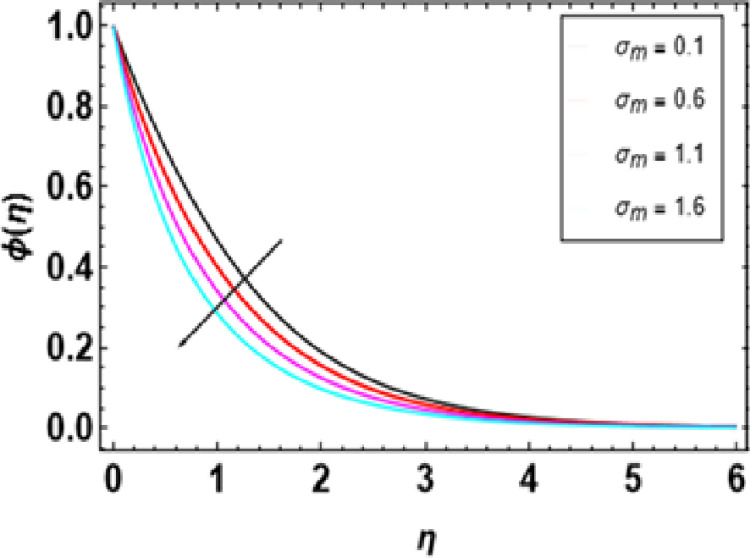
Outcomes of $\sigma_{m}$ on ϕ(η).

As the Schmidt number ascends, the concentration profiles show a gradual increment, as seen in Fig 19. Schmidt number measures the relative importance of momentum diffusion compared to mass diffusion in a fluid (solute diffusion). The higher the Schmidt number gets, the more significant viscosity becomes than diffusivity, which leads to the concentration profile becoming more pronounced such that it seems like nanoparticles are much more concentrated within the layer boundary of concentration. Therefore, there is a steeper gradient in concentration, which intensifies faster along the flow direction. This means that when compared to diffusivity, high viscosity hampers nanoparticle dispersion, so they can still be packed together so closely. Fig 20 shows how the nanoparticle volume fraction profile depends on the chemical reaction parameter for positive and negative values of this parameter. This can be seen by observing that the nanoparticle volume fraction decreases when the chemical reaction is of a building type but increases if it is destructive. With increasing nanoparticle volume fraction, nanofluids become more thermally conductive. As a result, this increased thermal conductivity thickens the concentration boundary layer. When the volume fraction parameter rises above zero, respectively, convective transport in a fluid will be supported better, leading to higher mass transfer rates or operations with higher concentrations.

**Fig 21:**
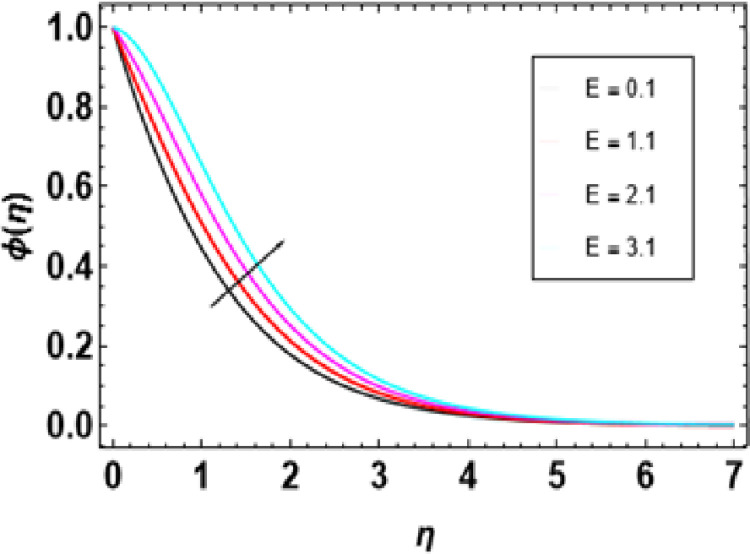
Outcomes of E onϕ(η).

Then, the diagram in Fig 21 shows the effect of activation energy on concentration profile if it is increased; this causes a steep increase in concentration profiles. This is based on how activation energy plays a part in chemical reactions. Activation energy (E) represents the least energy necessary for reactants to change into products during a chemical reaction. Reactant molecules must overcome this barrier to form product molecules; subsequently, the activation energy defines it. The breaking of bonds in reactants and initiating reactions depend on activation energy. When the activation energy is high, there will have to be a more significant barrier for the same ions participating in the bonds’ rupture before recombining to form new ones again, slowing down reactions. At higher E values, the reaction rate decreases, causing an increase in the concentration profile. This arises from lower chances that reactants will overcome such an obstacle and transform into products.

**Fig 22:**
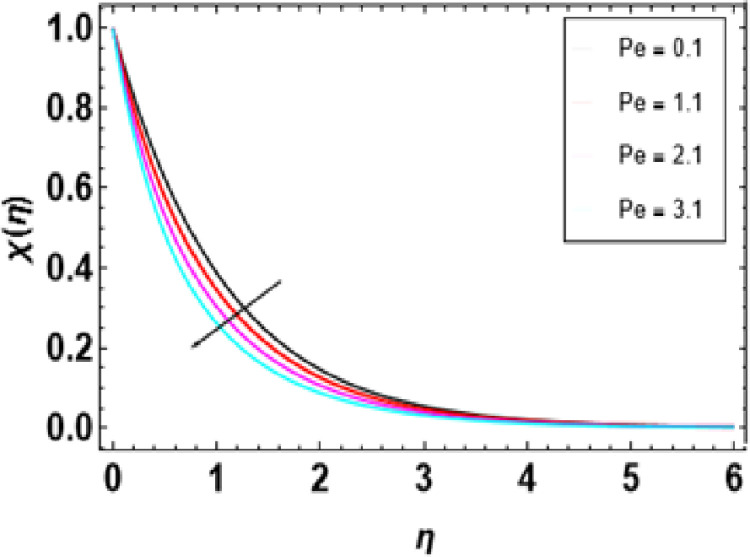
Outcomes of Pe on χ(η).

**Fig 23:**
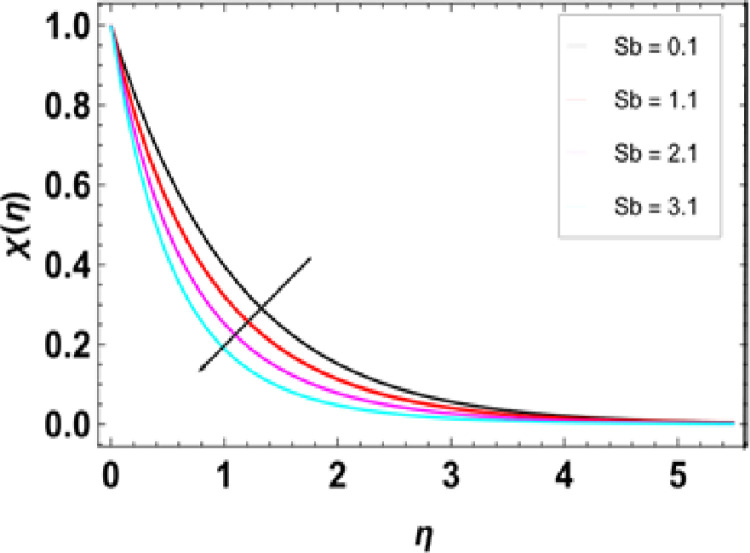
Outcomes of Sb on χ(η).

**Fig 24:**
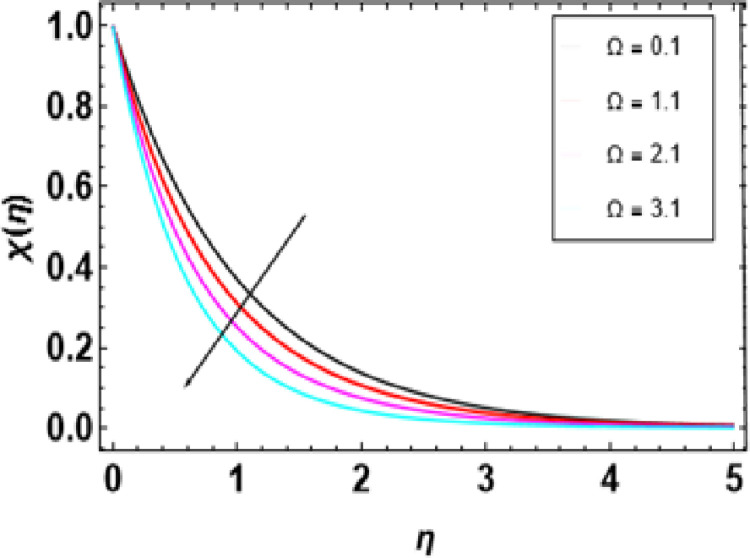
Outcomes of *Ω* on χ(η).

**Fig 25:**
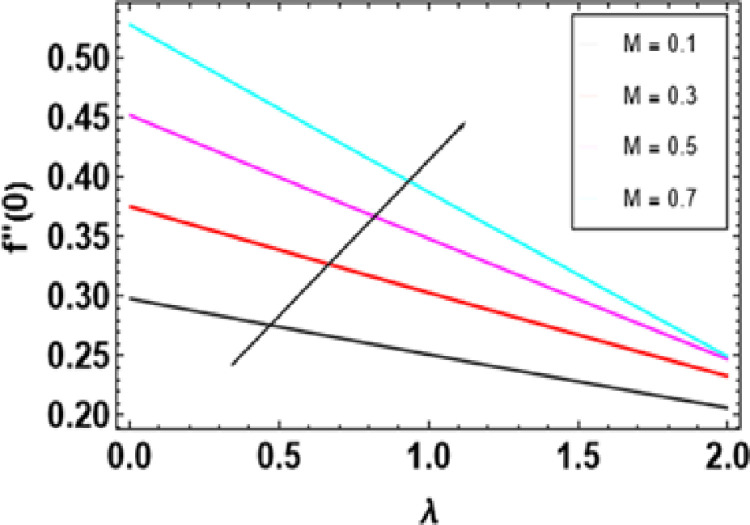
Variations of M and λ to f�(0).

**Fig 26:**
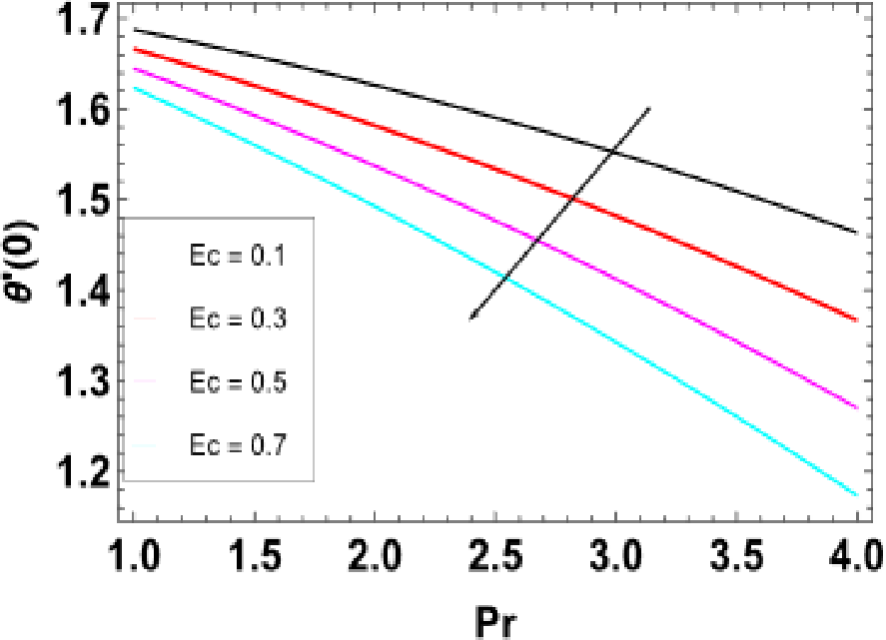
Variations of Pr and Ec to −θ'(0).

**Fig 27:**
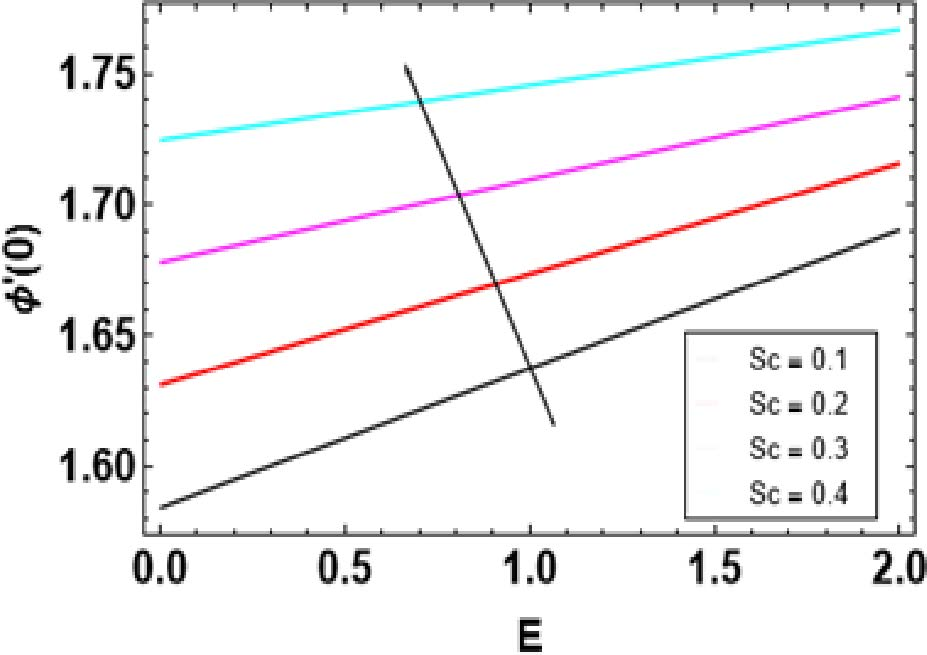
Variations of E and Sc to −ϕ ′ (0).

**Fig 28:**
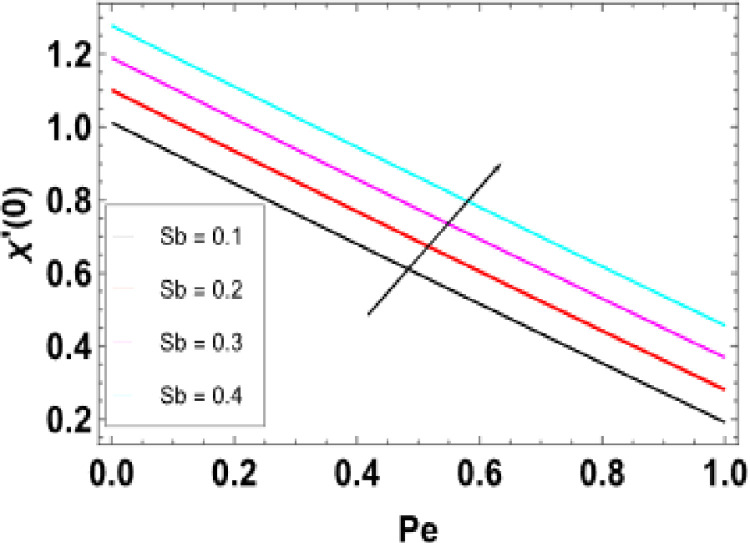
Variations of Sb and Pe to -χ�(0).

The microorganism profile is illustrated in Fig 22 and its dependence upon the dimensionless Peclet number. The decline in the microorganism profile can be observed due to an increase in different parameters, according to Fig 22. The Peclet number shows how much advection dominates mass diffusion. An increased Peclet number increases heat flux rates, thereby developing flow instability. However, in the interim period, with a rise in Peclet numbers, there is a reduction in the heat rate. Fig 23 shows that swimming microorganisms become less crowded as the Sb number increases. Schmidt number is a physical parameter in bioconvection that compares momentum diffusion with mass diffusion inside a fluid medium containing organisms and particles. This factor becomes helpful for grasping how materials circulate within such systems. High values of the Schmidt number indicate that mass transfer occurs at a greater rate than momentum transfer, meaning that fluid motion will be slower than the diffusion process. Fig 24 shows how bioconvection parameters affect motile density distribution. More inputs mean less concentration of motile bacteria as in fluid mobility. The nature of locomotion, for example, speed and sensitivity to environmental signals, can also alter the distribution of bacterial populations. Temperature fluctuations influence motile density distribution. The gradient of temperature is a key determinant of bioconvection. Changes in the viscosity of fluid may lead to altered patterns of bioconvection, which affects the distribution of motile density. Fig 25 depicts how different constraints, specifically M and **λ**, impact the drag force factor denoted as f’‘(0). The statement suggests that as the values of λ and M upsurge, the drag force factor also upsurges. Fig 26 illustrates how the Nusselt number, indicated as −θ'(0), varies with Pr and Ec. The statement implies that −θ'(0) declines with the upsurge in both Pr and Ec. Fig 27 demonstrates the association between the Sherwood number, designated as -ϕ’(0), and both E and Sc. The statement suggests that -ϕ’(0) upsurges with the rise in both E and Sc. In Fig 28, variations in the motile microorganism’s field, labelled as -χ’(0), are shown in response to constraints Pe and Lb changes. The findings suggest that as the values of Pe and Lb upsurge, there is a corresponding proportional rise in the gradient of motile microorganisms.

## 5.  Conclusion

An in-depth analysis examined the bioconvective thermal and mass transport on a Williamson nano liquid’s magnetohydrodynamic (MHD) boundary layer containing gyrotactic microbes. This liquid flows over an exponentially elongating surface in a porous medium. The study encompasses thermal emission, chemical response, and heat generation. Additionally, the rate of thermal transport exhibits rapid fluctuations attributed to variations in Joule heating, activation energy, and ohmic dissipation. The thermal transport rate, mass exchange, and mobile microorganism dynamics for the proposed model are computed and demonstrated both qualitatively and quantitatively. The Homotopy Analysis Method (HAM) technique resolves the problem.

- Thermophoresis and Brownian movement are significant in shaping temperature profile and the boundary layer thickness, which is crucial for heat distribution purposes.
- Thermal radiation decreases surface heat transfer, raises the temperature, lowers the system’s efficiency in transferring heat and changes its thermal behavior.
- Concentration gradient declines with increased Schmidt numbers and chemical reaction rates but improves with higher activation energy, thus implying that stronger reactions and higher Schmidt numbers reduce the gradient.
- Higher Peclet and smaller Schmidt numbers suggest less microbial spread in fluid, reducing microorganism dispersion.
- Increasing mixed convection enhances velocity, while bioconvection Rayleigh numbers at higher values and magnetic constraints slow the flow.
- The Homotopy Analysis Method (HAM) used in this research gives convergent solutions for all physical parameters, indicating its efficacy.

### Limitations

The study aims to use the Homotopy Analysis Method (HAM) to build theoretical models, which can fail to capture some of the realities of everyday life.

- The research assumes that nanoparticles are uniformly distributed and gyrotactic microorganisms behave ideally, which may not be practical, limiting the findings’ scope.
- It focuses on some parameters, such as thermal radiation and Joule heating, but fails to consider other aspects like external forces and changing geometries, which could influence flow dynamics.
- Whereas it concentrates on Williamson nanofluids, there is a possibility that non-Newtonian behavior may miss out completely, thereby compromising flow and heat transfer prediction accuracy.
- Mostly, they are analytical solutions based on results with few experimental investigations undertaken; hence, reliability concerns arise for actual practice.
- Besides describing gyrotactic microorganisms, this study does not fully account for their interaction complexity with fluid and other particles, thus making bioconvection processes incomplete.

### Future work

This study’s analytical results should be confirmed by future research through experiments so that the practicality of the findings can be improved.

- To MHD bioconvection for better understanding, future studies may vary parameters such as different nanoparticles, sheet geometries and external forces.
- More complex non-Newtonian fluid behaviors must be incorporated in further research to obtain deeper insights into flow and heat transfer beyond Williamson nanofluids.
- This will help to improve models for realistic scenarios of bioconvection by investigating how gyrotactic microorganisms interact with the fluid.
- For a more detailed analysis of flow patterns and thermal behaviors under various conditions, this research could include numerical simulations in the future.
- The researchers should have included temperature and pressure variations resulting from environmental factors for their findings to be relevant in real-life contexts.

**Table:** Nomenclature

Symbol	Name	Symbol	Name
M	Magnetic field Stricture	δ	Temperature distinction stricture
W	Williamson fluid stricture	Sc	Schmidt number
λ	Mixed convection stricture	Lb	Bio convection Lewis number
Nr	Buoyancy ratio stricture	Pe	Peclet number
T	Fluid Temperature	E	Dimensionless activation energy
Nt	Thermophoresis diffusion	$T_{w}$	Wall Temperature
Nb	Brownian motion factor	$T_{\infty }$	Temperature far away from the sheet
B	Dimensionless reaction rate	u,v	Velocity components
μ	Dynamic Velocity	$\sigma_{m}$	Bioconvection difference stricture
K	Radiation stricture	ν	Kinematic viscosity
$U_{w}$	Speed of wall	Rb	Bioconvection Rayleigh number

## References

[1] Ullah Awan A, Shah SAA, Ali B. Bio-convection effects on Williamson nanofluid flow with exponential heat source and motile microorganism over a stretching sheet. Chinese Journal of Physics. 2022;77:2795–810. doi: 10.1016/j.cjph.2022.04.002

[2] Raza R, Mabood F, Naz R, Abdelsalam SI. Thermal transport of radiative Williamson fluid over stretchable curved surface. Thermal Science and Engineering Progress. 2021;23:100887. doi: 10.1016/j.tsep.2021.100887

[3] Taj M, Salahuddin T. A three dimensional frictional flow study of Williamson fluid with chemical reaction. Materials Science and Engineering: B. 2023;291:116305. doi: 10.1016/j.mseb.2023.116305

[4] Khan U, Zaib A, Ishak A, Bakar SA, Animasaun IL, Yook S-J. Insights into the dynamics of blood conveying gold nanoparticles on a curved surface when suction, thermal radiation, and Lorentz force are significant: The case of Non-Newtonian Williamson fluid. Mathematics and Computers in Simulation. 2022;193:250–68. doi: 10.1016/j.matcom.2021.10.014

[5] Rafique K, Mahmood Z, , Khan U, Ali B, Awwad FA, et al. Numerical analysis of non-linear radiative Casson fluids containing CNTs having length and radius over permeable moving plate. Open Physics. 2024;22(1):2040013-28. doi: 10.1515/phys-2024-0013

[6] Avinash K, Sandeep N, Makinde OD, Animasaun IL. Aligned Magnetic Field Effect on Radiative Bioconvection Flow Past a Vertical Plate with Thermophoresis and Brownian Motion. DDF. 2017;377:127–40. doi: 10.4028/www.scientific.net/ddf.377.127

[7] Makinde OD, Animasaun IL. Thermophoresis and Brownian motion effects on MHD bioconvection of nanofluid with nonlinear thermal radiation and quartic chemical reaction past an upper horizontal surface of a paraboloid of revolution. Journal of Molecular Liquids. 2016;221:733–43. doi: 10.1016/j.molliq.2016.06.047

[8] Shashikumar NS, Madhu M, Sindhu S, Gireesha BJ, Kishan N. Thermal analysis of MHD Williamson fluid flow through a microchannel. International Communications in Heat and Mass Transfer. 2021;127:105582. doi: 10.1016/j.icheatmasstransfer.2021.105582

[9] Khan KA, Javed MF, Ullah MA, Riaz MB. Heat and Mass transport analysis for Williamson MHD nanofluid flow over a stretched sheet. Results in Physics. 2023;53:106873. doi: 10.1016/j.rinp.2023.106873

[10] Saleem M, Hussain M. Impression of nonlinear radiation and Stefan blowing on the magneto cross nano-Williamson fluid above exponentially stretching sheet. Results in Engineering. 2023;17:100864. doi: 10.1016/j.rineng.2022.100864

[11] Alqahtani AM, Rafique K, Mahmood Z, Al-Sinan BR, Khan U, Hassan AM. MHD rotating flow over a stretching surface: The role of viscosity and aggregation of nanoparticles. Heliyon. 2023;9(11):e21107. doi: 10.1016/j.heliyon.2023.e21107 37928015 PMC10623290

[12] Zafar M, Mahmood K, Rafique A, Adnan U, Khan S, Jubair FA, et al. Significance of shape factor on magnetohydrodynamic buoyancy thin film flow of nanofluid over inclined sheet with slip condition: irreversibility analysis. Modern Physics Letters B. 2023;245:0335–48.

[13] SivaSankari M, Eswara Rao M, Khan W, Alshehri MH, Eldin SM, Iqbal S. Analytical analysis of the double stratification on Casson nanofluid over an exponential stretching sheet. Case Studies in Thermal Engineering. 2023;50(1):103492–506. doi: 10.1016/j.csite.2023.103492

[14] Iqbal MA, Usman M, Allehiany FM, Hussain M, Khan KA. Rotating MHD Williamson nanofluid flow in 3D over exponentially stretching sheet with variable thermal conductivity and diffusivity. Heliyon. 2023;9(11):e22294. doi: 10.1016/j.heliyon.2023.e22294 38027644 PMC10679479

[15] Amjad M, Ahmed K, Akbar T, Muhammad T, Ahmed I, Alshomrani AS. Numerical investigation of double diffusion heat flux model in Williamson nanofluid over an exponentially stretching surface with variable thermal conductivity. Case Studies in Thermal Engineering. 2022;36 102231. doi: 10.1016/j.csite.2022.102231

[16] Mahmood Z, Rafique K, Khan U, Muhammad T, , Alballa T, et al. Heat transfer in radiative hybrid nanofluids over moving sheet with porous media and slip conditions: Numerical analysis of variable viscosity and thermal conductivity. Materials Today Communications. 2024;40 109664. doi: 10.1016/j.mtcomm.2024.109664

[17] Mahmood Z, Rafique K, Khan U, , Abd El-Rahman M, Alharbi R. Analysis of mixed convective stagnation point flow of hybrid nanofluid over sheet with variable thermal conductivity and slip Conditions: A Model-Based study. International Journal of Heat and Fluid Flow. 2024;106 109296. doi: 10.1016/j.ijheatfluidflow.2024.109296

[18] Salahuddin T, Fatima G, Awais M, Khan M, Al Awan B. Adaptation of nanofluids with magnetohydrodynamic Williamson fluid to enhance the thermal and solutal flow analysis with viscous dissipation: A numerical study. Results in Engineering. 2024;21:101798. doi: 10.1016/j.rineng.2024.101798

[19] Taj M, Salahuddin T. Analysis of viscously dissipated three-dimensional flow of Williamson fluid with nonlinear radiation and activation energy. Alexandria Engineering Journal. 2023;76:595–607. doi: 10.1016/j.aej.2023.06.043

[20] Maaitah H, Olimat AN, Quran O, Duwairi HM. Viscous dissipation analysis of Williamson fluid over a horizontal saturated porous plate at constant wall temperature. International Journal of Thermofluids. 2023;19:100361. doi: 10.1016/j.ijft.2023.100361

[21] Sadighi S, Jabbari M, Afshar H, Danesh Ashtiani HA. MHD heat and mass transfer nanofluid flow on a porous cylinder with chemical reaction and viscous dissipation effects: Benchmark solutions. Case Studies in Thermal Engineering. 2022;40:102443. doi: 10.1016/j.csite.2022.102443

[22] Rafique K, Mahmood Z, Adnan A, Ali B, Khan U, Muhammad T, et al. Entropy analysis of Hall effect with variable viscosity and slip conditions on rotating hybrid nanofluid flow over nonlinear radiative surface. Materialstoday communication. 2024;39:109167–86.

[23] Khan U, Rafique K, Mahmood Z. Significance of unsteady rotating flow of nanofluid with nanoparticles aggregation and impacts of slip conditions and variable viscosity. Numerical Heat Transfer, Part A: Applications, (2024), 1–28.

[24] Kumar A, Tripathi R, Singh R, Chaurasiya VK. Simultaneous effects of nonlinear thermal radiation and Joule heating on the flow of Williamson nanofluid with entropy generation. Physica A: Statistical Mechanics and its Applications. 2020;551:123972. doi: 10.1016/j.physa.2019.123972

[25] Khan MI, Qayyum S, Hayat T, Khan MI, Alsaedi A. Entropy optimization in flow of Williamson nanofluid in the presence of chemical reaction and Joule heating. International Journal of Heat and Mass Transfer. 2019;133:959–67. doi: 10.1016/j.ijheatmasstransfer.2018.12.168

[26] Abbas A, Khan A, Abdeljawad T, Aslam M. Numerical simulation of variable density and magnetohydrodynamics effects on heat generating and dissipating Williamson Sakiadis flow in a porous space: Impact of solar radiation and Joule heating. Heliyon. 2023;9(11):e21726. doi: 10.1016/j.heliyon.2023.e21726 38027754 PMC10643495

[27] Rafique K, Mahmood Z, , Khan U, Muhammad T, El-Rahman MA, et al. Numerical investigation of entropy generation of Joule heating in non-axisymmetric flow of hybrid nanofluid towards stretching surface. Journal of Computational Design and Engineering. 2024;11(2):146–60. doi: 10.1093/jcde/qwae029

[28] Hussain M, Jahan S, Ranjha QA, Ahmad J, Jamil MK, Ali A. Suction/blowing impact on magneto-hydrodynamic mixed convection flow of Williamson fluid through stretching porous wedge with viscous dissipation and internal heat generation/absorption. Results in Engineering. 2022;16:100709. doi: 10.1016/j.rineng.2022.100709

[29] Mishra P, Kumar D, Reddy YD, Goud BS. MHD Williamson micropolar fluid flow pasting a non-linearly stretching sheet under the presence of non linear heat generation/ absorption. Journal of the Indian Chemical Society. 2023;100(1):100845. doi: 10.1016/j.jics.2022.100845

[30] Cui J, Razzaq R, Farooq U, Khan WA, Farooq FB, Muhammad T. Impact of non-similar modeling for forced convection analysis of nano-fluid flow over stretching sheet with chemical reaction and heat generation. Alexandria Engineering Journal. 2022;61(6):4253–61. doi: 10.1016/j.aej.2021.09.045

[31] Makinde OD, Animasaun IL. Bioconvection in MHD nanofluid flow with nonlinear thermal radiation and quartic autocatalysis chemical reaction past an upper surface of a paraboloid of revolution. International Journal of Thermal Sciences. 2016;109:159–71. doi: 10.1016/j.ijthermalsci.2016.06.003

[32] Srinu A, Reddy KS, Amar N. Radiation and inclined magnetic field effects on Williamson fluid flow above a stretching sheet in the existence of velocity, thermal, and concentration slips. Partial Differential Equations in Applied Mathematics. 2024;9:100611. doi: 10.1016/j.padiff.2023.100611

[33] Wang F, Tarakaramu N, Sivakumar N, Narayana PVS, Harish Babu D, Ramalingam S. Three dimensional nanofluid motion with convective boundary condition in presents of nonlinear thermal radiation via stretching sheet. Journal of the Indian Chemical Society. 2023;100(2):100887. doi: 10.1016/j.jics.2023.100887

[34] Puneeth V, Manjunatha S, Makinde OD, Gireesha BJ. Bioconvection of a Radiating Hybrid Nanofluid Past a Thin Needle in the Presence of Heterogeneous–Homogeneous Chemical Reaction. Journal of Heat Transfer. 2021;143(4):042502-042518. doi: 10.1115/1.4049844

[35] Ramzan M, Rehman S, Junaid MS, Saeed A, Kumam P, Watthayu W. Dynamics of Williamson Ferro-nanofluid due to bioconvection in the portfolio of magnetic dipole and activation energy over a stretching sheet. International Communications in Heat and Mass Transfer. 2022;137:106245. doi: 10.1016/j.icheatmasstransfer.2022.106245

[36] Hussain Z, Khan WA, Ali M, Waqas M, Ashraf IM. Chemically reactive magneto-bioconvection 3D flow of radiative williamson nanofluid containing oxytactic moment of microorganisms. Tribology International. 2023;189:108934. doi: 10.1016/j.triboint.2023.108934

[37] Yousaf A, Imran M, Yasmin S, Ali MR. Numerical assessment of bioconvection in MHD Prandtl nanofluid with gyrotactic motile microorganisms with bio-fuel applications. Case Studies in Thermal Engineering. 2023;52:103639. doi: 10.1016/j.csite.2023.103639

[38] Eswara Rao M, Siva Sankari M, Nagalakshmi Ch, Rajkumar S, On the Role of Bioconvection and Activation Energy for MHD-Stretched Flow of Williamson and Casson Nanofluid Transportation across a Porous Medium Past a Permeable Sheet. Journal of Nanomaterial. 2023;(2023):1-12.

[39] Asjad MI, Zahid M, Inc M, Baleanu D, Almohsen B. Impact of activation energy and MHD on Williamson fluid flow in the presence of bioconvection. Alexandria Engineering Journal. 2022;61(11):8715–27. doi: 10.1016/j.aej.2022.02.013

[40] Shoaib M, Hayat T, Ali N, Sajid M, Javed T. Non-similar analysis of entropy optimized chemically reactive flow of Williamson fluid with activation energy. Journal of Magnetism and Magnetic Materials. 2023;586:171173. doi: 10.1016/j.jmmm.2023.171173

[41] Zhang X, Yang D, Ur Rehman MI, Mousa AA, Hamid A. Numerical simulation of bioconvection radiative flow of Williamson nanofluid past a vertical stretching cylinder with activation energy and swimming microorganisms. Case Studies in Thermal Engineering. 2022;33:101977. doi: 10.1016/j.csite.2022.101977

[42] Khan WA, Makinde OD. MHD nanofluid bioconvection due to gyrotactic microorganisms over a convectively heat stretching sheet. International Journal of Thermal Sciences. 2014;81:118–24. doi: 10.1016/j.ijthermalsci.2014.03.009

[43] Bourchak Ramzi Othman M, Kada B, Hussain I, AliPasha A, AzeemKhan W, Tabrez M, Juhany K. Significance of gyrotactic microorganism and bioconvection analysis for radiative Williamson fluid flow with ferromagnetic nanoparticles. Thermal Science and Engineering Progress. 2023;39:101732-46.

[44] Bhatti MM, Arain MB, Zeeshan A, Ellahi R, Doranehgard MH. Swimming of Gyrotactic Microorganism in MHD Williamson nanofluid flow between rotating circular plates embedded in porous medium: Application of thermal energy storage. Journal of Energy Storage. 2022;45:103511. doi: 10.1016/j.est.2021.103511

[45] Rana BMJ, Arifuzzaman SM, Islam S, Reza-E-Rabbi Sk, Al-Mamun A, Mazumder M, et al. Swimming of microbes in blood flow of nano-bioconvective Williamson fluid. Thermal Science and Engineering Progress. 2021;25:101018. doi: 10.1016/j.tsep.2021.101018

